# Properties, Pharmacology, and Pharmacokinetics of Active Indole and Oxindole Alkaloids in *Uncaria* Hook

**DOI:** 10.3389/fphar.2021.688670

**Published:** 2021-07-14

**Authors:** Hirotaka Kushida, Takashi Matsumoto, Yasushi Ikarashi

**Affiliations:** Tsumura Kampo Research Laboratories, Kampo Research & Development Division, Tsumura & Co., Ibaraki, Japan

**Keywords:** *Uncaria* HooK, indole alkaloid, oxindole alkaloid, pharmacokinetics, absorption, metabolism, distribution, excretion

## Abstract

*Uncaria* Hook (UH) is a dry stem with hook of *Ucaria* plant and is contained in Traditional Japanese and Chinese medicine such as yokukansan, yokukansankachimpihange, chotosan, Gouteng-Baitouweng, and Tianma-Gouteng Yin. UH contains active indole and oxindole alkaloids and has the therapeutic effects on ailments of the cardiovascular and central nervous systems. The recent advances of analytical technology led to reports of detailed pharmacokinetics of UH alkaloids. These observations of pharmacokinetics are extremely important for understanding the treatment’s pharmacological activity, efficacy, and safety. This review describes properties, pharmacology, and the recently accumulated pharmacokinetic findings of UH alkaloids, and discusses challenges and future prospects. UH contains major indole and oxindole alkaloids such as corynoxeine, isocorynoxeine, rhynchophylline, isorhynchophylline, hirsuteine, hirsutine, and geissoschizine methyl ether (GM). These alkaloids exert neuroprotective effects against Alzheimer’s disease, Parkinson’s disease, and depression, and the mechanisms of these effects include anti-oxidant, anti-inflammatory, and neuromodulatory activities. Among the UH alkaloids, GM exhibits comparatively potent pharmacological activity (e.g., agonist activity at 5-HT_1A_ receptors). UH alkaloids are absorbed into the blood circulation and rapidly eliminated when orally administered. UH alkaloids are predominantly metabolized by Cytochrome P450 (CYP) and converted into various metabolites, including oxidized and demethylated forms. Regarding GM metabolism by CYPs, a gender-dependent difference is observed in rats but not in humans. Several alkaloids are detected in the brain after passing through the blood–brain barrier in rats upon orally administered. GM is uniformly distributed in the brain and binds to various channels and receptors such as the 5-HT receptor. By reviewing the pharmacokinetics of UH alkaloids, challenges were found, such as differences in pharmacokinetics between pure drug and crude drug products administration, food-influenced absorption, metabolite excretion profile, and intestinal tissue metabolism of UH alkaloids. This review will provide readers with a better understanding of the pharmacokinetics of UH alkaloids and their future challenges, and will be helpful for further research on UH alkaloids and crude drug products containing UH.

## Introduction

**FIGURE 1:**
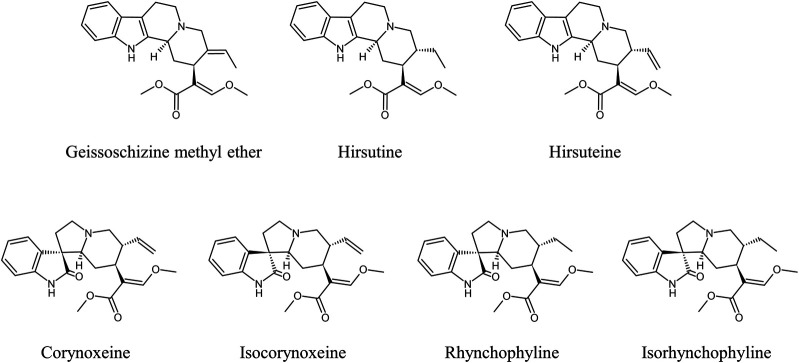
Chemical structure of the main indole and oxindole alkaloids in UH.

*Uncaria* Hook (UH) is a dry stem and hook of *Uncaria* plant in the family Rubiaceae (Ndagijimana et al., 2013; Liang et al., 2020). Many botanical origins have been identified for UH (Zhang et al., 2015), but three origins, namely *Uncaria rhynchophylla* (Miq.) Miq., *Uncaria sinensis* (Oliv.) Havil., and *Uncaria macrophylla* Wall., are listed in the Japanese Pharmacopoeia. UH contains several indole and oxindole alkaloids such as corynoxeine (CX), isocorynoxeine (ICX), rhynchophylline (RP), isorhynchophylline (IRP), hirsuteine (HTE), hirsutine (HTI), and geissoschizine methyl ether (GM) (Figure 1), which have similar pharmacological neuroprotective effects against Alzheimer’s disease, Parkinson’s disease, and depression, and the mechanisms of these effects include anti-oxidant, anti-inflammatory, and neuromodulatory activities (Pengsuparp et al., 2001; Ueda et al., 2011; Ndagijimana et al., 2013; Qi et al., 2014; Yang et al., 2020).

UH alkaloids are also the active components of traditional Japanese Kampo medicines such as yokukansan (YKS) (Ikarashi et al., 2018), yokukansankachimpihange (YKSCH) (Takiyama et al., 2019), and chotosan (CTS) (Yuzurihara et al., 2002) containing UH as a crude drug. These Kampo medicines have been approved by the Japanese Ministry of Health, Labour and Welfare (MHLW), and they are indicated for the treatment of neurosis, insomnia, and irritability and night crying in children, in addition to headache and hypertension (Ministry of Health, Labour, and Welfare MHLW, 2016). In China, Yi-Gan San (YGS), which has the same composition as YKS (Gai et al., 2014), Gouteng-Baitouweng consisting of the hook and stem of *Uncaria rhynchophylla* (Miq.) Miq. (*Gouteng* in Chinese) and the root of *Pulsatilla chinensis* (Bunge) Regel (*Baitouweng* in Chinese) (Tian et al., 2018), and Tianma-Gouteng Yin decoction/granules consisting of 11 herbal materials (Liu et al., 2015; Zhang H. et al., 2019) are known as UH-containing crude drug products. Gouteng-Baitouweng is clinically used for convulsion, a neurological symptom associated with Parkinson’s disease (Ishikawa et al., 2006). Tianma-Gouteng Yin is one of the traditional Chinese medicines prescribed to treat Parkinson’s disease like symptoms such as tremor and paralysis (Chua et al., 2012).

Many studies were carried out to date on the mechanism of pharmacological action of UH alkaloids. It was experimentally shown that CX have inhibitory effects on aortic vascular smooth muscle cells (VSMC) proliferation (Kim et al., 2008) and to promote the clearance of alpha-synuclein (α-syn) by induced-autophagy (Chen et al., 2014), and ICX has vasorelaxantion (Li et al., 2017). *In vivo* studies demonstrated that RP and/or IRP have anti-depressant and neuroprotective effects (Fu et al., 2014; Sakakibara et al., 1999). Their mechanisms included the enhancement of α-syn clearance by induced-autophagy (Li et al., 2017) and effects through central dopaminergic system (Sakakibara et al., 1999) or the central monoaminergic neurotransmitter system (Xian et al., 2017). HTI and HTE, like other UH alkaloids, were also demonstrated to possess a protective effect against neuronal death (Kanno et al., 2014). Basic *in vivo* and *in vitro* studies illustrated that GM alleviates experimental anxiety, aggressiveness, dementia, allodynia, and vasoconstriction (Watanabe et al., 2003; Matsumoto et al., 2013; Mizoguchi and Ikarashi, 2017; Ikarashi et al., 2018). Multiple actions such as serotonergic (Pengsuparp et al., 2001; Nishi et al., 2012: Ueki et al., 2013), dopaminergic (Ueda et al., 2011; Ishida et al., 2016), and adrenergic neurotransmission (Nakagawa et al., 2012), as well as neuroprotection (Kawakami et al., 2011; Kanno et al., 2014; Sun et al., 2016), are considered as the mechanisms underlying the pharmacological effects of GM (Ikarashi and Mizoguchi, 2016; Ikarashi et al., 2018).

Although much pharmacological evidence had been accumulated for UH-containing crude drug products (Egashira et al., 2001; Murakami et al., 2005; Zhao et al., 2005; Ito et al., 2013; Ikarashi and Mizoguchi, 2016; Tabuchi et al., 2017; Jiang et al., 2019), few studies had been performed on their pharmacokinetics until recently. In particular, there was little information on the pharmacokinetics of GM compared to that of essential *Uncaria* alkaloids such as RP, IRP, CX, ICX, HTE, and HTI (Nakazawa et al., 2006; Wang et al., 2010a; Wang et al., 2010b; Wang et al., 2014a; Wang et al., 2014b; Wang et al., 2017; Zhang et al., 2017; Chen et al., 2020). However, as multiple and potent pharmacological activities of GM have become apparent, with breakthroughs of analytical technologies, pharmacokinetic information has also gradually been accumulated, such as its absorption (Ma et al., 2014; Han et al., 2019), exposure to circulation blood (Kushida et al., 2013; Gai et al., 2014; Kitagawa et al., 2015; Zhang H. et al., 2019), hepatic metabolism (Kushida et al., 2015; Matsumoto et al., 2016; Tian et al., 2018; Kushida et al., 2021), blood–brain barrier (BBB) permeability (Imamura et al., 2011), brain distribution (Mizoguchi et al., 2014; Matsumoto et al., 2020), and urinary excretion (Gai et al., 2014; Matsumoto et al., 2018).

Pharmacokinetic findings such as the absorption, distribution, metabolism, and excretion of active indole and oxindole alkaloids in the body after the oral administration of UH-containing crude drug products are extremely important and essential information for interpreting their pharmacological activity, efficacy, and safety. Therefore, we aimed to review data on the pharmacokinetics of UH alkaloids accumulated in recent decades and to improve the reader’s understanding of them. We also considered the challenges and future prospects of UH alkaloid pharmacokinetic studies.

## Methodology for Collecting Literature

We first retrieved studies published over the past 21 years (2000–2021) using the search terms “geissoschizine methyl ether,” “corynoxeine,” “isocorynoxeine,” “rhynchophylline,” “isorhynchophylline,” “hirsuteine,” and “hirsutine” in PubMed, PMC, and ScienceDirect. From the identified articles, 55 English articles describing the pharmacokinetics and pharmacology of UH alkaloids were selected for this review.

## Properties of UH Alkaloids

Seven major active indole and oxindole alkaloids are present in UH (Figure 1). GM has a molecular formula of C_22_H_26_N_2_O_3_, a molecular weight of 366.45 g/mol, acid dissociation constant (pKa) of 8.25, and octanol–water partition coefficient (LogP) of 3.445. CX and ICX are epimers with a molecular formula of C_22_H_26_N_2_O_4_ (382.45 g/mol), pKa of 8.03, and LogP of 0.989. RP and IRP are also epimers with a molecular formula of C_22_H_26_N_2_O_4_ (384.47 g/mol), pKa of 8.49, and LogP of 1.633. HTE is a structural isomer of GM with a molecular formula of C_22_H_26_N_2_O_3_ (366.45 g/mol), pKa of 8.37, and LogP of 2.875. HTI with a molecular formula of C_22_H_28_N_2_O_3_ (368.47 g/mol), pKa of 8.83, and LogP of 3.703 (https://scifinder-n.cas.org/). To date, no transformation between GM and other UH alkaloids has been reported, although epimerization between RP and IRP and between CX and ICX has been described (Qi et al., 2015; Wang et al., 2017).

The contents of UH alkaloids in five plant origins (*Uncaria rhynchophylla* (Miq.) Miq., *Uncaria sinensis* (Oliv.) Havil., *Uncaria macrophylla* Wall., *Uncaria hirsuta* Havil, and *Uncaria sessilifructus* Roxb.) has been measured (Hou et al., 2018). Concerning alkaloids derived from *Uncaria rhynchophylla* (Miq.) Miq., seven indole and oxindole alkaloids were present (0.0020–0.2096%). In particular, the GM content ranged from 0.0214 to 0.2096%. However, GM was not present in the remaining four plants. No other alkaloids were present in *Uncaria sinensis* (Oliv.) Havil. and *Uncaria hirsuta* Havil. The contents of UH alkaloids in crude drug products have also been measured. The GM content in YKS extract is reportedly 0.0054% (54 μg/g) or 0.014% (Nishi et al., 2012; Kushida et al., 2021). The contents of other UH ingredients in the YKS extract comprises of CX, ICX, RP, IRP, HTE, and HTI at 0.026, 0.009, 0.027, 0.005, 0.015, and 0.013%, respectively, which are similar to the GM contents (Nishi et al., 2012).

## Pharmacological Effects and Mechanisms

The main indole and oxindole alkaloids in UH have been revealed to have neuroprotective effects against Alzheimer’s disease, Parkinson’s disease, and depression, and the mechanisms of these effects include anti-oxidant, anti-inflammatory, and neuromodulatory activities (Pengsuparp et al., 2001; Ueda et al., 2011; Ndagijimana et al., 2013; Yang et al., 2020).

The proliferation of VSMCs induced by damage to the arterial intima is an important etiological factor in vascular proliferative diseases, such as atherosclerosis and restenosis. Kim et al. (2008) reported that CX has inhibitory effects on platelet-derived growth factor (PDGF)-BB–induced rat aortic VSMC proliferation. Pretreatment of VSMCs with CX (5–50 μM) for 24 h resulted in significant decrease in the cell number (25.0–88.0%). CX (5–50 μM) significantly inhibited DNA synthesis in VSMCs induced by 50 ng/ml PDGF-BB (32.8–88.0%). Preincubation of VSMCs with CX significantly inhibited the activation of extracellular signal-regulated kinases 1/2 induced by PDGF-BB. The major component of Lewy bodies in patients with Parkinson’s disease is α-syn, and it is believed that the impairments of the autophagy–lysosomal pathway are related to α-syn accumulation. CX-induced autophagy, which was paralleled by increased expression of the lysosomal enzyme cathepsin D in different neuronal cell lines, including N_2a_ and SHSY-5Y cells. Furthermore, by inducing autophagy, CX promoted the clearance of wild-type and A53T α-syn in inducible Pheochromocytoma (PC12) cells. CX-induced autophagy was mediated by the protein kinase B/mammalian target of rapamycin (Akt/mTOR) pathway (Chen et al., 2014). It has been reported that ICX, the epimer of CX, has vasorelaxant effects (Li et al., 2017). The mechanism of vasorelaxation involves the inhibition of external Ca^2+^ influx *via* L-type calcium channels and intracellular Ca^2+^ release *via* α_1A_ adrenergic receptors in VSMCs as well as the involvement of K channels.

The major neuropathological features of Alzheimer’s disease include the extracellular accumulation of amyloid-β (Aβ) peptide and intracellular neurofibrillary tangles composed of hyperphosphorylated tau protein. Oral RP treatment at a dose of 50 mg/kg effectively reduced the activity of Ephrin type-A receptor 4 (EphA4) and blocked EphA4-dependent signaling pathways, which negatively regulates synaptic plasticity in the hippocampal neurons, thereby restoring the amyloid precursor protein gene and presenilin 1 gene in transgenic mice (Fu et al., 2014). Furthermore, RP and IRP (1–100 µM) exerted neuroprotective effects against Aβ_25-35_–induced neuronal toxicity in PC12 cells by inhibiting intracellular calcium overload and tau protein hyperphosphorylation (Xian et al., 2012). It has been reported that IRP promotes the autophagy-induced clearance of α-syn in neuronal cells. IRP (0.3 μM) inhibited 1-methyl-4-phenyl pyridinium^+^-induced apoptosis in PC12 cells (Li et al., 2017). In another study, IRP (6.25–25 μM) induced autophagy in N_2a_, SH-SY5Y, and PC12 cell lines as well as in primary cortical neurons, *via* the mTOR pathway, thereby promoting the clearance of various forms of α-syn in neuronal cells (Lu et al., 2012). UH alkaloids have been reported to exert anti-depressant effects. Sakakibara et al. (1999) reported that CX (30 mg/kg), IRP (100 mg/kg), and GM (100 mg/kg) markedly decreased locomotor activity through the central dopaminergic system. In another study, IRN (10–40 mg/kg) exerted an anti-depressant–like effect in mice in the forced swimming and tail suspension tests by modulating the central monoaminergic neurotransmitter system and inhibiting the activity of monoamine oxidase A (Xian et al., 2017).

HTI and HTE, like other UH alkaloids, have also been demonstrated to possess a protective effect against neuronal death (Kanno et al., 2014). Kanno et al. (2014) evaluated the neuroprotective effects of HTI, HTE, and GM on glutamate-induced cell death with 3-(4,5-dimethylthiazol-2-yl)-2,5-diphenyl-tetrazolium bromide (MTT) staining. Cell survival against control cells exposed to glutamate alone was significantly increased with the addition of GM (3 µM), HTE (10 µM), and HTI (10 µM). These components enhanced gene expressions of system Xc^−^ subunits xCT and 4F2hc, and also ameliorated the glutamate-induced decrease in glutathione (GSH) levels. These results suggest that the cytoprotective effect of HTI, HTE, and GM may be due to the suppression of GSH reduction by enhancing system Xc^−^.

GM reportedly exerts multiple effects, such as serotonergic, dopaminergic, and adrenergic neurotransmission, as well as neuroprotection. Using Chinese hamster ovary cells artificially expressing the 5-HT_1A_ receptor, the competitive binding assay and (^3^H)8-hydroxy-2-(di-n-propylamino) tetralin binding assay of seven major alkaloids in UH have been investigated. Among the seven alkaloids, only GM exhibited strong binding to 5-HT_1A_ receptors (IC_50_: 0.904 µM), acting as a partial agonist (Nishi et al., 2012). The effect of YKS and UH-containing alkaloids on the L-DOPA-derived dopamine production has been investigated using RIN 14B cells that are 5-HT synthesizing cells. YKS and certain alkaloids (CX and GM) inhibited catechol-*O*-methyltransferase activity (2000 µg/ml YKS: 83.1%, 100 nmol/ml CX: 72.4%, 100 nmol/mL GM: 65.1%) and significantly increased dopamine production (Ishida et al., 2016). Competitive radioligand and (^35^S) GTPγS binding assays have revealed that YKS and GM had specific binding affinity for and antagonist activity against the α_2A_-adrenoceptor (IC_50_: approximately 300 μg/ml and 3 µM, respectively), being approximately 10 times stronger than that of hirusutin and hirustein (Nakagawa et al., 2012). Oral administration of 1 g/kg YKS improved aggressive behavior and restored social behavior in isolated mice, and oral administration of 150 μg/kg GM, equivalent to 1 g/kg YKS, showed similar pharmacological effects (Nishi et al., 2012).

## Detection and Quantification Methods

Measuring UH alkaloids levels is essential for elucidating their pharmacokinetics. Pharmacokinetic studies of UH alkaloids have been progressing along with the development of analytical technologies. To date, various analytical methods for detecting, identifying, and quantifying UH alkaloids have been developed and selectively used according to the purpose of the study (Table 1). For example, high–performance liquid chromatography (HPLC) has been used as a fingerprint analysis to support the quality control of several Japanese Kampo medicines containing UH (Tsuji et al., 2014; Nakatani et al., 2017) and as a quantitative analysis of UH alkaloids including GM (Hou et al., 2018). Furthermore, liquid chromatography with tandem mass spectrometry (LC–MS/MS) has been utilized to quantify UH alkaloids concentrations in biological samples, enabling the highly selective and sensitive detection of them. In addition, LC–MS/MS has also been applied to identify and quantify UH alkaloids and their metabolites in plasma, brain, urine, or bile samples from rats and mice after the oral administration of UH-containing crude drug products or pure UH alkaloids (Kushida et al., 2013; Wu et al., 2013; Gai et al., 2014; Zhao et al., 2016b; Tian et al., 2018; Zhang H. et al., 2019; Han et al., 2019; Takiyama et al., 2019; Chen et al., 2020; Matsumoto et al., 2020). In addition, LC–MS/MS has been applied to simultaneously quantify alkaloids in extracts from several *Uncaria* species for standardization and quality control and to conduct chemical classification of the *Uncaria* genus (Wang H.-B. et al., 2014). An *in vitro* autoradiography assay using (^3^H)GM has been performed to determine GM-specific binding sites on brain sections (Mizoguchi et al., 2014). Mass spectrometry imaging (MSI) using matrix-assisted laser desorption ionization (MALDI) (Giordano et al., 2016; Matsumoto et al., 2017) and desorption electrospray ionization (DESI) (Fernandes et al., 2016), which are powerful tools for visualizing the distributions of biological molecules or metabolites in tissue sections, have been applied to obtain images of the distribution of GM in rat brains (Matsumoto et al., 2020).

**TABLE 1:** Lower limit of quantification and calibration range of UH alkaloids in various analytical methods.

Analytical method	Matrix	Compond	LLOQ (ng/mL or ng/mg)	Calibration range (ng/ml or ng/mg)	References
HPLC-UV	Methanol	CX	441.3	1,400–56,500	Hou et al. (2018)
		ICX	405.4	800–31,100	
		RP	339.0	1,000–40,500	
		IRP	400.0	1,000–40,200	
		HTE	512.0	5,100–102,400	
		HTI	635.3	7,300–110,300	
		GM	466.7	7,000–105,000	
LC-MS/MS	Plasma	CX	0.3	0.3–50	Kushida et al. (2013)
		ICX and RP	0.05	0.05–50	
		IRP and HTE	0.1	0.1–50	
		HTI	0.05	0.05–50	
		GM	0.03	0.03–50	
	Brain	CX, ICX, RP, IRP, HTE, HTI, and GM	0.5	0.5–250	
	Plasma	RP and HTI	2.5	2.5–50	Wu et al. (2013)
	Plasma	ICX	0.05	0.05–19.80	Gai et al. (2014)
		RP	0.05	0.05–21.40	
	Plasma	CX and ICX	20	20–2,000	Zhao et al. (2016b)
	Plasma	CX, ICX, RP, and IRP	0.1	0.3–1,000	Tian et al. (2018)
		GM	0.2	0.3–1,000	
	Plasma	HTE and HTI	1	1–200	Han et al. (2019)
	Plasma	GM	0.025	0.0250–50.0	Takiyama et al. (2019)
	Plasma	CX, ICX, RP, IRP, HTE, and HTI	1	1–1,000	Chen et al. (2020)
MSI-DESI	Brain	GM	6,250	6,250–25,000	Matsumoto et al. (2020)

LLOQ: Lower limit of quantification.

## Pharmacokinetics

Traditional Japanese and Chinese medicines are generally complex products consisting of two or more crude drugs. Therefore, a single crude drug contains several components with different chemical structures. These structures may affect the properties of the coexisting components through physical and chemical interactions. As a result, the properties of the components of crude drug products might differ significantly from those of the pure components. For this reason, pharmacokinetic studies using crude drug products have limitations. However, because crude drug products are often used in clinical practice rather than individual ingredients, pharmacokinetic studies using crude drug products are useful for studying and understanding the pharmacology of the ingredients under conditions that reflect their clinical use. Conversely, pharmacokinetic studies using individual components reveal the properties of the pure components. Ideally, pharmacokinetic studies of components in crude drugs should be performed using both crude drug mixtures and pure components, as this will provide a better understanding of the properties of the components. Therefore, in this section, we have reviewed the pharmacokinetic properties of UH alkaloids, focusing on GMs, separately for pharmacokinetic studies using UH-containing crude drug products and pure UH alkaloids.

### Absorption

Recently, the pharmacokinetics and bioavailability of six UH alkaloids were reported in mouse blood (Chen et al., 2020). In particular, the bioavailabilities of CX, ICX, RP, IRP, HTE, and HTI in mice treated at a dose of 5 mg/kg orally or 1 mg/kg intravenously were 27.3, 32.7, 49.4, 29.5, 51.0, and 68.9%, respectively. In another study, the bioavailabilities of HTI and HTE in rats at the same dose were 4.4 and 8.21%, respectively (Han et al., 2019).

The ability of RP to permeate Caco-2 cell monolayers has been investigated (Ma et al., 2014). RP permeated the monolayer with velocities of 2.76 × 10^−6^–5.57 × 10^–6^ cm/s from the apical side to the basolateral side and 10.68 × 10^−6^–15.66 × 10^–6^ cm/s from the basolateral side to the apical side. The ability of RP to permeate cells was increased by verapamil, a P-glycoprotein (P-gp) inhibitor, and rhodamine 123, a P-gp substrate. Furthermore, RP induced the expression of P-gp in Caco-2 cells. These results indicate that P-gp may be involved in the low permeability and absorption of RP. In another study, Wang et al. (2017) investigated RP and IRP membrane permeability and efflux by P-gp using *in situ* single-pass intestinal perfusion with and without verapamil. The intestinal perfusion assay illustrated that IRP possessed higher intestinal permeability than RP, and co-perfusion with verapamil could affect the absorption of RP but not IRP. These results suggest that RP, but not IRP, is a substrate of P-gp.

### Exposure to Blood

#### Administration of UH-Containing Crude Drug Products

Most pharmacokinetic studies of GM have been performed after the oral administration of crude drug products (Kushida et al., 2013; Matsumoto et al., 2018; Takiyama, et al., 2019) (Table 2). Although the absolute bioavailability of GM has not been verified, many studies revealed that GM in the intestinal lumen following oral administration is certainly absorbed into portal vein blood and transferred to circulating blood. The pharmacokinetics of circulating plasma GM was first reported in male rats orally administered YKS (0.25, 1, and 4 g/kg) (Kushida et al., 2013); GM was detected in the plasma after YKS administration, and the plasma maximum concentration (*C*_max_) increased in a dose-dependent manner (0.261–1.980 ng/ml) with a nearly similar time to reach the peak plasma concentration (*t*_max_ = 0.42–0.67 h). The apparent half-life (*t*_1/2_) in three different YKS dose groups were similar (1.5–2.0 h), indicating that plasma GM was rapidly eliminated. The area under the plasma concentration–time curve (AUC) increased dose-dependently in the range of 0.441–6.79 ng h/mL. In male mice, GM was detected (approximately 2.5 ng/ml) in plasma 1 h after oral YKS administration (1 g/kg), and it had mostly disappeared after 8–24 h (Matsumoto et al., 2018). In addition to YKS, GM has also been detected in the plasma of rats orally administered Tianma-Gouteng Yin granules at doses of 2.5 and 5 g/kg (Zhang H. et al., 2019).

**TABLE 2:** Pharmacokinetic studies of UH alkaloids.

Drug type	Drug/Route/Animal	Outcome	References
Crude drug product	YKS (0.25, 1, 4 g/kg), oral, male rats	AUC_0-∞_, *C*_max_, *t*_max_, and *t*_1/2_ in plasma of 0.0544–0.656 ng h/ml, 0.0759–0.274 ng/ml, 0.42–1.0 h, and 1.4 h, respectively, for RP; 0.149–1.59 ng h/ml, 0.160–0.381 ng/ml, 0.50–0.83 h, and 3.4 h, respectively, for HTI; 0.0829–2.08 ng h/ml, 0.125–0.529 ng/ml, 0.50–2.0 h, and 1.9–3.6 h, respectively, for HTE; and 0.441–6.79 ng h/ml, 0.261–1.98 ng/ml, 0.42–0.67 h, and 1.5–2.0 h, respectively, for GM.	Kushida et al. (2013)
		AUC_0-∞_, *C*_max_, *t*_max_, and *t*_1/2_ in brain of 2.90 ng h/g, 1.18 ng/g, 0.50 h, and 3.4 h (4 g/kg), respectively	
	YKS (1 g/kg), oral, male mice	GM concentration of about 2.5 ng/ml at 1 h	Matsumoto et al. (2018)
	YKS (1, 4 g/kg), oral, female rats	GM concentrations in plasma of 2.1 and 9.0 ng/ml, respectively	Imamura et al. (2011)
		GM concentrations in brain of 1.6 and 5.9 ng/ml, respectively	
	YKS (1 g/kg), oral, female rats	*C*_max_, *t*_max_, *t*_1/2α_, *t*_1/2β_, and AUC of 12.3 ng/ml, 0.508 h, 0.194 h, 31.1 h, and 29.3 ng h/ml, respectively	Takiyama et al. (2019)
	YKS (4 g/kg), oral, female rats	*C*_max_, *t*_max_, *t*_1/2_, *k*_e_, and AUC_0-∞_ of 8.46 ng/ml, 1.17 h, 2.14 h, 0.33 h^−1^, and 33.7 ng h/ml, respectively	Kushida et al. (2021)
	YKS (2.5, 5.0, or 7.5 g/day), oral, male and female humans	*C*_max_, AUC_0-last_, *t*_1/2_, and *t*_max_ of 0.650–1.98 ng/ml, 1.18–4.81 ng h/ml, 1.72–1.95 h, and 0.500 h, respectively, for GM and of 0.138–0.450 ng/ml, 0.277–1.50 ng h/ml, 2.47–3.03 h, and 0.975–1.00 h, respectively, for HTE.	Kitagawa et al. (2015)
	YGS (9.1 g/kg), oral, rats	AUC, *C*_max_, *t*_max_, *t*_1/2_, MRT, CL/F, and V/F were 64.70 ng h/ml, 9.43 ng/ml, 4.76 h, 8.68 h, 7.25 h, 2.72 L h/kg, and 39.79, respectively, for RP and 41.02 ng h/ml, 3.89 ng/ml, 1.79 h, 9.11 h, 7.82 h, 6.65 L h/kg, and 83.73 L/kg, respectively, for ICX.	Gai et al. (2014)
	Gouteng-Baitouweng (25 g/kg), oral, rats	AUCs of RP, IRP, CX, ICX, and GM in portal vein plasma were 2083.2, 647.0, 767.3, 2,237.4, and 41.1 ng h/ml, respectively.	Tian et al. (2018)
		AUCs of RP, IRP, CX, and ICX in systemic plasma were 349.6, 51.0, 35.1, and 265.8 ng h/ml, respectively	
Pure drug	GM (5 mg/kg), intravenous, rats	One-compartment model with *k*_e_ of 0.4 h^−1^, a *t*_1/2_ of 1.8 h, a CL_total_ of 1.7 L/h/kg, and Vd of 4.4 L/kg	Matsumoto et al. (2020)
	CX, ICX, RP, IRP, HTI, and HTE (5 mg/kg), oral, mice	AUCs of CX, ICX, RP, IRP, HTI, and HTE were 260.9, 293.1, 416.7, 209.2, 899.7, and 511.3 ng h/ml, respectively, *C*_max_ were 374.8, 373.2, 508.0, 305.3, 524.5, and 360.8 ng/ml, respectively, MRT were 0.9, 1.6, 1.7, 1.2, 1.8, and 1.6 h; respectively, t1/2 were 0.7, 1.6, 4.4, 2.5, 2.0, and 2.4 h, respectively; CL/F were 20.2, 18.2, 11.6, 24.7, 5.6, and 9.8 L/h/kg, respectively; and V/F were 19.3, 45.7, 60.1, 84.5, 16.8, and 36.1 L/kg, respectively	Chen et al. (2020)
	CX, ICX, RP, IRP, HTI, and HTE (1 mg/kg), intravenous, mice	AUCs of CX, ICX, RP, IRP, HTI, and HTE were 191.4, 179.2, 168.6, 142.0, 261.3, and 200.7 ng h/ml, respectively, *C*_max_ were 230.7, 221.2, 199.3, 184.6, 222.6, and 188.4 ng/ml, respectively, MRT were 0.2, 0.3, 0.5, 0.2, 0.9, and 0.9 h; respectively, t1/2 were 0.5, 3.2, 2.2, 0.6, 1.1, and 2.2 h, respectively; CL were 6.2, 6.1, 6.2, 7.4, 4.4, and 5.2 L/h/kg, respectively; and V were 4.4, 28.7, 20.0, 6.8, 8.3, and 15.9 L/kg, respectively	
	HTI, and HTE, (5 mg/kg), oral, rats	AUC of HTI and HTE were 70.8 and 70.3, respectively, Cmax were 21.9 and 17.8 ng/ml, MRT were 3.6 and 4.4 h, respectively, t1/2 were 1.8 and 3.5 h, respectively, CL/F were 73.2 and 74.0 L/h/kg, respectively, and V/F were 189.4 and 359.0 L/kg, respectively	Han et al. (2019)
	HTI, and HTE, (1 mg/kg), intravenous, rats	AUC of HTI and HTE were 322.9 and 173.3, respectively, *C*_max_ were 97.9 and 72.5 ng/ml, MRT were 5.0 and 3.1 h, respectively, t1/2 were 2.6 and 4.1 h, respectively, CL were 3.1 and 5.7 L/h/kg, respectively, and V were 11.9 and 34.9 L/kg, respectively	

GM has also been detected in female rat plasma after the oral administration of YKS (Imamura et al., 2011), but its detailed pharmacokinetics has only recently been reported (Takiyama et al., 2019). In that report, similarly as observed in male rats, plasma GM concentrations in female rats rapidly increased (*C*max = 12.3 ng/ml, *t*max = 0.508 h) after the oral administration of YKS (1.0 g/kg), and then the compound immediately disappeared (*t*1/2α = 0.194 h, *t*1/2β = 31.1 h) over 24 h. AUC was 29.3 ng h/mL. When *C*_max_ and AUC were compared between female (Takiyama et al., 2019) and male rats (Kushida et al., 2013) administered the same dose of YKS (1 g/kg), the plasma GM concentration was much higher in female rats than in male rats. These findings suggested gender differences in plasma GM concentrations in rats. Indeed, we recently demonstrated that a significant gender difference in the rat plasma pharmacokinetics of GM by revealing that *C*_max_ and AUC of plasma GM in female rats were approximately 4-fold higher than those in male rats after oral administration of the same dose of YKS (Kushida et al., 2021). The causes of the gender differences in rat plasma GM pharmacokinetics are discussed in the “Mmetabolism by Cytochrome P450 in the Liver” section.

In humans, a randomized crossover study examined the pharmacokinetics of several active components including GM using 21 healthy Japanese volunteers who orally received YKS (2.5, 5.0, or 7.5 g/day). *C*_max_ of GM increased in a dose-dependent manner over the range of 0.650–1.98 ng/ml with a similar *t*_max_ (0.500 h) across the groups. *t*_1/2_ was similar among the groups, ranging from 1.72 to 1.95 h. Consequently, AUC increased in a dose-dependent manner, ranging from 1.18 to 4.81 ng h/mL (Kitagawa et al., 2015). The rapid rise and disappearance of plasma GM after oral YKS administration in humans were similar to those in rodents, but unlike rats, no gender differences in GM plasma pharmacokinetics were observed. This result suggests that humans probably have no gender differences in GM metabolism, although more clinical evidence needs to be accumulated in the future.

Similar to the findings for GM, the blood concentrations of other UH alkaloids during the administration of crude drug products have also been investigated. We have studied the pharmacokinetics of UH alkaloids in plasma after oral YKS administration (0.25, 1, and 4 g/kg) in male rats (Kushida et al., 2013). In addition to GM, RP, HTI, and HTE were detected in rat plasma, whereas CX, ICX, and IRP were not detected. RP, HTI, and HTE appeared rapidly in the circulating blood and disappeared rapidly, and the plasma concentration–time curves were similar to those of GM. AUC, *C*max, *t*max, and *t*1/2 were 0.0544–0.656 ng h/ml, 0.0759–0.274 ng/ml, 0.42–1.0 h, and 1.4 h, respectively, for RP; 0.149–1.59 ng h/ml, 0.160–0.381 ng/ml, 0.50–0.83 h, and 3.4 h, respectively, for HTI; and 0.0829–2.08 ng h/ml, 0.125–0.529 ng/ml, 0.50–2.0 h, and 1.9–3.6 h, respectively, for HTE. Gai et al. (2014) measured ICX and RP levels in rat plasma after the oral administration of 9.1 g/kg YGS, which has the same composition as YKS, *via* targeted analysis using LC-MS/MS and calculated their pharmacokinetic parameters. AUC, *C*_max_, *t*_max_, *t*_1/2_, the mean residence time (MRT), clearance rate/bioavailability (CL/F), and the apparent distribution volume/bioavailability (V/F) were 64.70 ng h/ml, 9.43 ng/ml, 4.76 h, 8.68 h, 7.25 h, 2.72 L h/kg, and 39.79, respectively, for RP and 41.02 ng h/ml, 3.89 ng/ml, 1.79 h, 9.11 h, 7.82 h, 6.65 L h/kg, and 83.73 L/kg, respectively, for ICX. Regarding other UH-containing crude drug products, the pharmacokinetics of CX, ICX, RP, IRP, and GM in systemic and portal vein plasma following the oral administration of Gouteng-Baitouweng (25 g/kg) to rats was reported (Tian et al., 2018). All five UH alkaloids were detected in portal vein plasma, whereas all UH alkaloids except GM were detected in systemic plasma. The AUCs of RP, IRP, CX, ICX, and GM in portal vein plasma were 2083.2, 647.0, 767.3, 2,237.4, and 41.1 ng h/ml, respectively. Furthermore, those of RP, IRP, CX, and ICX in systemic plasma were 349.6, 51.0, 35.1, and 265.8 ng h/ml, respectively. The AUCs were much lower in systemic plasma than in portal vein plasma. These results suggest that UH alkaloids are metabolized in the liver. In addition, GM was not detected in systemic plasma. The GM concentration in portal vein plasma is much lower than that of other alkaloids, suggesting that the GM content in Gouteng-Baitouweng is low. It has been reported that the contents of indole and oxindole alkaloids in UH depend on its botanical origin and hook size (Hou et al., 2018).

#### Administration of Pure UH Alkaloids

As described previously, the pharmacokinetics of GM following the oral administration of YKS has been well studied, whereas information regarding its pharmacokinetics following the administration of GM alone is limited (Table 2). Matsumoto et al. (2020) reported the plasma concentrations vs. time profile following the intravenous injection of GM and found that the profile well fitted to a one-compartment model, and the pharmacokinetic parameters obtained in their model analysis were as follows: elimination rate constant of 0.4 h–1, *t*1/2 of 1.8 h, total CL of 1.7 L/h/kg, and V of 4.4 L/kg.

Conversely, several pharmacokinetic studies using pure UH alkaloids (excluding GM) have been reported (Han et al., 2019; Chen et al., 2020). Chen et al. (2020) determined the pharmacokinetic parameters of six indole and oxindole alkaloids after oral and intravenous administrations at doses of 5 and 1 mg/kg, respectively, in mice. In the case of oral administration, MRTs of CX, ICX, RP, IRP, HTI, and HTE were 0.9, 1.6, 1.7, 1.2, 1.8, and 1.6 h; respectively, the *t*_1/2_ times were 0.7, 1.6, 4.4, 2.5, 2.0, and 2.4 h, respectively; the CL/F values were 20.2, 18.2, 11.6, 24.7, 5.6, and 9.8 L/h/kg, respectively; and the V/F values were 19.3, 45.7, 60.1, 84.5, 16.8, and 36.1 L/kg, respectively. The pharmacokinetic parameters of HTI and HTE in rats following oral and intravenous administrations were calculated by Han et al. (2019). MRT (3.6 and 4.4 h) and *t*_1/2_ (1.8 and 3.5 h) of HTI and HTE in rats after oral administration were not significantly different from those in mice, but CL/F (73.2 and 74.0 L/h/kg) and V/F (189.4 and 359.0 L/kg) were approximately 10-fold larger. CL and V of HTI and HTE in rats after intravenous administration were almost the same as those in mice; specifically, the CL values of HTI and HTE were 4.4 and 5.2 L/h/kg, respectively, in mice and 3.1 and 5.7 L/h/kg, respectively, in rats. Similarly, the V values of HTI and HTE were 8.3 and 15.9 L/kg, respectively, in mice and 11.9 and 34.9 L/kg, respectively, in rats.

### Metabolism

#### Liver First-Pass Effect

When an herbal medicine or crude drug product is orally administered, its constituents are first absorbed into the portal vein blood *via* intestinal absorption and subjected to a first-pass effect in the liver. Subsequently, the compounds enter systemic blood, which carries them to the target tissue.

Table 3 showed detailed information of the metabolism studies of UH alkaloids. Tian et al. (2018) performed the simultaneous determination of five *Uncaria* alkaloids [an indole alkaloid GM and four oxindole alkaloids (RP, IRP, CX, and ICX)] in portal vein plasma, liver, systemic plasma, and brain samples after the oral administration of Gouteng-Baitouweng in male rats. All five alkaloids were detected in portal vein plasma and the liver. However, GM was not detected in systemic plasma. Although the four other alkaloids were detected in systemic plasma, their AUCs were significantly lower than those in portal vein plasma, i.e., plasma before hepatic metabolism. These results suggest that the liver first-pass effect plays a critical role in the pharmacokinetics of *Uncaria* alkaloids including GM.

**TABLE 3:** Metabolism studies of UH alkaloids.

Study type	Model/Subject	Drug/Route	Outcome	References
Liver first pass	Male rats	Gouteng-Baitouweng (25 g/kg), Oral	GM was detected in portal vein plasma, but not in systemic plasma	Tian et al. (2018)
	Male rats	Tianma-Gouteng granule (2.5 and 5 g/kg), Oral	GM and acidic or reduced/demethylated metabolites of GM were detected in plasma	Zhang et al. (2019b)
*In vitro* metabolism	Rat and human liver microsomes	GM	GM was metabolized into at least 13 metabolites including hydroxylated, dehydrogenated, hydroxylated/dehydrogenated, demethylated, and hydrated forms	Kushida et al. (2015)
	Human liver microsomes and recombinant human CYPs, RAF method	GM	GM was metabolized by CYP3A4 (61.3%), CYP2C19 (23.5%), and CYP2D6 (15.2%)	Matsumoto et al. (2016)
	Rat liver S9 and microsomes, recombinant rat CYPs	GM	CYP1A1, CYP2C6, CYP2C11, CYP2D1, and CYP3A2 were involved in GM metabolism	Kushida et al. (2021)
	Rat liver microsomes	IRP	25 metabolites produced by oxidation, hydrolysis, reduction, demethylation, hydroxylation, and dehydrogenation	Wang et al. (2016a)
	Rat liver microsomes	RP and IRP	Hydroxylation at the A-ring was the major metabolic pathway for RP	Wang et al. (2017)
			Oxidation at the C-ring was the major metabolic pathway for IRP	
	recombinant rat CYPs	ICX	CYP2C19 and CYP2D6 were involved in the production of 18,19-dehydrocorynoxinic acid, and CYP3A4 was involved in the production of 5-oxo ICX.	Zhao et al. (2016b)
	Rat liver microsomes, specific inhibitors	IRP	CYP1A1/2, CYP2C, and CYP2D, but not CYP3A, are involved in the 10- and 11-hydroxylation of IRP.	Wang et al. (2010a)
	Rat liver microsomes, specific inhibitors	RP	CYP1A1/2, CYP2C, and CYP2D, but not CYP3A, are involved in the 10- and 11-hydroxylation of RP	Wang et al. (2010b)
	Rat liver microsomes, specific inhibitors	HTI and HTE	CYP2C is involved in the 10- and 11-hydroxylation of HTI and THE	Nakazawa et al. (2006)
*In vivo* metabolism	Male rats	Tianma-Gouteng Yin granule (2.5 and 5 g/kg), Oral	GM and acidic or reduced/demethylated metabolites of GM were detected in plasma.	Zhang et al. (2019b)
			Corynoxeinic acid, isocorynoxeinic acid, rhynchophyllinic acid, isorhynchophyllinic acid, and hirsuteinic acid, and their 22-O-β-glucuronides were detected in plasma and bile, respectively	
	Rats	CX (0.105 mM/kg), oral	10- and 11-Hydroxy CX have been isolated from urine and faces, and 10-hydroxy CX 10-O-β-D-glucuronide and 11-hydroxy CX 11-O-β-D-glucuronide were isolated from the bile	Wang et al. (2014b)
	Rats	ICX (40 mg/k), oral	10- and 11-Hydroxy ICX, 10-hydroxy ICX 10-O-β-D-glucuronide and 11-hydroxy ICX 11-O-β-D-glucuronide were isolated from the bile	Chen et al. (2014)
	Rats	ICX (40 mg/kg), oral	11-hydroxy ICX, 5-oxoisocorynoxeinic acid-22-O-β-D-glucuronide, 10-hydroxy ICX, 17-O-demethyl-16,17-dihydro-5-oxo ICX, 5-oxoisocorynoxeinic acid, 21-hydroxy-5-oxo ICX, oxireno[18, 19]-5-oxo ICX, 18,19-dehydrocorynoxinic acid, 18,19-dehydrocorynoxinic acid B, CX, ICX-N-oxide, and CX-N-oxide were detected in urine	Qi et al. (2015)
	Rats	ICX (40 mg/kg), oral	18, 33, and 18 metabolites produced by hydrolysis, oxidation, isomerization, demethylation, epoxidation, reduction, glucuronidation, hydroxylation, and N-oxidation were detected in plasma, urine, and bile, respectively	Zhao et al. (2016a)
	Rats	ICX (40 mg/kg), oral	8,19-dehydrocorynoxinic acid, 18,19-dehydrocorynoxinic acid B, 5-oxoisocorynoxeinic acid-22-O-glucuronide, 17-O-demethyl-16,17-dihydro-5-oxo ICX, 5-oxoisocorynoxeinic acid, and 5-oxoisorhynchophyllic acid were identified in plasma	Zhao et al. (2016b)
	Rats	IRP (20 mg/kg), oral	10- and 11-Hydroxy IRP were isolated from urine and feces.	Wang et al. (2010a)
			10- and 11-Hydroxy IRP-β-O-glucuronides were isolated from bile	
	Rats	RP (37.5 mg/kg), oral	10- and 11-Hydroxy RP were isolated from urine and feces.	Wang et al. (2010b)
			10- and 11-Hydroxy RP-β-O-glucuronides were isolated from bile	
	Rats	IRP (20 mg/kg), oral	47, 21, and 18 metabolites of IRP were identified in rat urine, plasma, and liver, respectively	Wang et al. (2016a)
	Rats	HTI (20 mg/kg), oral	67 metabolites by hydroxylation, dehydrogenation, oxidation, N-oxidation, hydrolysis, reduction, and glucuronide conjugation	Wang et al. (2016b)
	Rats	HTI and HTE (50 mg/kg), oral	11-hydroxy HTE-11-O-β-D-glucuronide, 11-hydroxy HTE, 11-hydroxy HTI-11-O-β-D-glucuronide, and 11-hydroxy HTI from the bile and urine	Nakazawa et al. (2006)

Zhang H. et al. (2019) identified the absorbed *Uncaria* alkaloids and their metabolites in the suborbital vein plasma and bile of male rats orally administered Tianma-Gouteng granules. Indole alkaloids (GM, HTE, and HTI) and oxindole alkaloids (RP, IRP, CX, and ICX) were detected in plasma along with their acidic or reduced/demethylated metabolites. Concerning the oxindole alkaloids, their glucuronidated metabolites were additionally detected in plasma and bile, suggesting that oxindole alkaloids, but not indole alkaloids, are excreted in bile as glucuronidated metabolites. However, the possibility of the biliary excretion of indole alkaloids cannot be dismissed by this study alone because the content of indole alkaloids might be low in Tianma-Gouteng granules. In addition to this finding, Nakazawa et al. (2006) detected 11-hydroxyhirsuteine-11-*O*-*β*-D-glucuronide or 11-hydroxyhirsutine-11-*O*-*β*-D- glucuronide was detected in the bile and urine of rats orally administered a high dose (50 mg/kg) of the GM-like indole alkaloid HTE or HTI, respectively. The study suggested that the glucuronides were predominantly excreted into bile rather than urine (Nakazawa et al., 2006). Therefore, the biliary excretion of GM should be examined in more detail in the future.

#### *In Vitro* Metabolites

GM metabolites were identified or estimated from fragment ion patterns obtained by LC–MS/MS analysis after incubating GM with rat and human liver microsomes, and their molecular structures were proposed based on the characteristics of their precursor and product ions, as well as their chromatographic retention times (Kushida et al., 2015). At least 13 GM metabolites were found in the incubation sample compared to the blank sample. The 13 confirmed metabolites were 2 demethylation (M1-1 and M1-2), 1 dehydrogenation (M2-1), 3 oxidation/dehydrogenation (M3-1, M3-2, and M3-3), 3 oxidation (M4-1, M4-2, and M4-3), 2 water adduction (M5-1 and M5-2), 1 di-demethylation (M6-1), and 1 water adduction/oxidation metabolites (M7-1). No difference could be observed between rat and human liver microsome-produced versions of these metabolites.

In addition to GM, the metabolites of RP and IRP have also been investigated *via in vitro* metabolism studies using liver microsomes (Wang J. et al., 2016; Wang et al., 2018). After rat liver microsomes were incubated with RP or IRP, five RP metabolites and three IRP metabolites were found to react with the A- and C-rings. Among metabolic pathways including oxidation, hydroxylation, N-oxidation, and dehydrogenation, hydroxylation at the A-ring was the major metabolic pathway for RP, whereas oxidation at the C-ring was the major metabolic pathway for IRP (Wang et al., 2018). Furthermore, Wang J. et al. (2016) also found that after incubation of rat liver microsomes with IRP, 25 metabolites were produced through six metabolic pathways: oxidation, hydrolysis, reduction, demethylation, hydroxylation, and dehydrogenation. Of these, hydrolysis, hydroxylation, and oxidation are the main metabolic pathways of IRP.

#### *In Vivo* Metabolites

To date, several GM metabolites have been identified *via in vivo* studies. When Tianma-Gouteng Yin granules were administered orally to rats at doses of 2.5 and 5 g/kg, demethylated GM was identified in plasma and bile (Zhang H. et al., 2019). Furthermore, 18-hydroxy-GM was detected in 8-h urine samples from mice orally administered with YGS (Gai et al., 2014). This GM metabolite is potentially predicted to be M4-3. The hydroxylated GM metabolites were also detected by DESI–MSI in the brain tissue from intravenously GM-injected rats (Matsumoto et al., 2020). Furthermore, studies using the RAF method suggested that the production of hydroxylated GM metabolites in humans was the main reaction (Matsumoto et al., 2016). From these results, the main GM metabolite form is potentially inferred to be hydroxylated. As described in the “Liver first-pass effect” section, hydroxylated GM metabolites are thought to be subsequently excreted in the urine as glucuronide conjugates. However, few GM metabolite-related studies have been performed so far in biological samples, such as plasma, urine, and bile. In order to fully understand the pharmacological activity and safety of GM, it would be indispensable to identify its metabolites.

Contrarily, several studies investigated the metabolites of other UH alkaloids. Corynoxeinic acid, isocorynoxeinic acid, rhynchophyllinic acid, isorhynchophyllinic acid, and hirsuteinic acid (demethylated metabolites), as well as their 22-*O*-*β*-glucuronides, have been detected in plasma and bile after the oral administration of Tianma-Gouteng Yin granules (2.5 and 5 g/kg) to rats (Zhang H. et al., 2019).

10- and 11-Hydroxy CX have been isolated from urine and faces, and 10-hydroxy CX 10-*O*-*β*-D-glucuronide and 11-hydroxy CX 11-*O*-*β*-D-glucuronide were isolated from the bile of rats orally administered pure CX (Wang W. et al., 2014). Concerning ICX, the epimer of CX, 10- and 11-hydroxy ICX and their 10- and 11-*β*-*O*-glucuronides have been isolated from the excreta and bile of rats who were administered pure ICX, similarly as observed for CX (Chen et al., 2014). In another study, 11-hydroxy ICX, 5-oxoisocorynoxeinic acid-22-*O*-*β*-D-glucuronide, 10-hydroxy ICX, 17-*O*-demethyl-16,17-dihydro-5-oxo ICX, 5-oxoisocorynoxeinic acid, 21-hydroxy-5-oxo ICX, oxireno[18, 19]-5-oxo ICX, 18,19-dehydrocorynoxinic acid, 18,19-dehydrocorynoxinic acid B, CX, ICX-N-oxide, and CX-N-oxide were detected in rat urine following oral ICX administration (Qi et al., 2015). After oral ICX administration, 18, 33, and 18 metabolites produced by hydrolysis, oxidation, isomerization, demethylation, epoxidation, reduction, glucuronidation, hydroxylation, and N-oxidation were detected in plasma, urine, and bile, respectively (Zhao et al., 2016a), and 18,19-dehydrocorynoxinic acid, 18,19-dehydrocorynoxinic acid B, 5-oxoisocorynoxeinic acid-22-*O*-glucuronide, 17-*O*-demethyl-16,17-dihydro-5-oxo ICX, 5-oxoisocorynoxeinic acid, and 5-oxoisorhynchophyllic acid were the main metabolites of ICX in plasma (Zhao et al., 2016b).

As 10- and 11-Hydroxy RP and IRP have been isolated from urine and feces, and their 10- and 11-*β*-*O*-glucuronides were isolated from bile in *in vivo* studies using rats administered pure RP or IRP (Wang et al., 2010a; Wang et al., 2010b). In another study, 47, 21, and 18 metabolites were identified in rat urine, plasma, and liver, respectively, after the oral administration of pure IRP. Seven metabolic pathways, namely dehydrogenation, oxidation, hydrolysis, reduction, demethylation, hydroxylation, and glucuronidation were involved in the metabolism. Among them, dehydrogenation, hydrolysis, hydroxylation, and oxidation were considered the main metabolic pathways according to the *in vivo* and *in vitro* metabolic profiles (Wang J. et al., 2016).

Nakazawa et al. (2006) isolated 11-hydroxy HTE-11-*O*-*β*-D-glucuronide, 11-hydroxy HTE, 11-hydroxy HTI-11-*O*-*β*-D-glucuronide, and 11-hydroxy HTI from the bile and urine of rats treated with HTE and HTI. In addition, Wang et al. (2016b) found that after oral administration to rats, HTI was converted to 67 metabolites by hydroxylation, dehydrogenation, oxidation, N-oxidation, hydrolysis, reduction, and glucuronide conjugation.

#### Metabolism by CYPs in the Liver

As mentioned in the previous section, GM is metabolized into at least 13 metabolites including hydroxylated, dehydrogenated, hydroxylated/dehydrogenated, demethylated, and hydrated forms, as described by an *in vitro* study using rat and human liver microsomes. A subsequent study identified CYP isoforms predominantly involved in GM metabolism in human liver microsomes. The relative contribution ratio of each CYP isoform was estimated using the relative activity factor method, revealing that the majority of GM (61.3%) is dehydrogenated and/or hydroxylated by CYP3A4, 23.5% is demethylated or hydrated by CYP2C19, and the remaining 15.2% is demethylated or hydroxylated by CYP2D6 (Matsumoto et al., 2016).

In the “Exposure to Blood” section, we described gender differences of GM pharmacokinetics in rats but not in humans. Generally, pharmacological and pharmacokinetic studies of drugs in rodents are performed to predict efficacy and safety in humans. To date, humans are known to differ from model animals in terms of the composition, expression, and catalytic activity of drug-metabolizing enzymes and CYP isoforms. Particularly, CYP1A, CYP2C, CYP2D, and CYP3A subfamilies have exhibited considerable interspecies differences with respect to catalytic activity (Martignoni et al., 2016). Furthermore, gender differences in CYP2C (2C7, 2C11, 2C12, 2C13) and CYP3A isoforms (3A2, 3A9, 3A18) have been reported in rats (Martignoni et al., 2016). The question was whether these CYP isoforms are involved in GM metabolism.

**FIGURE 2:**
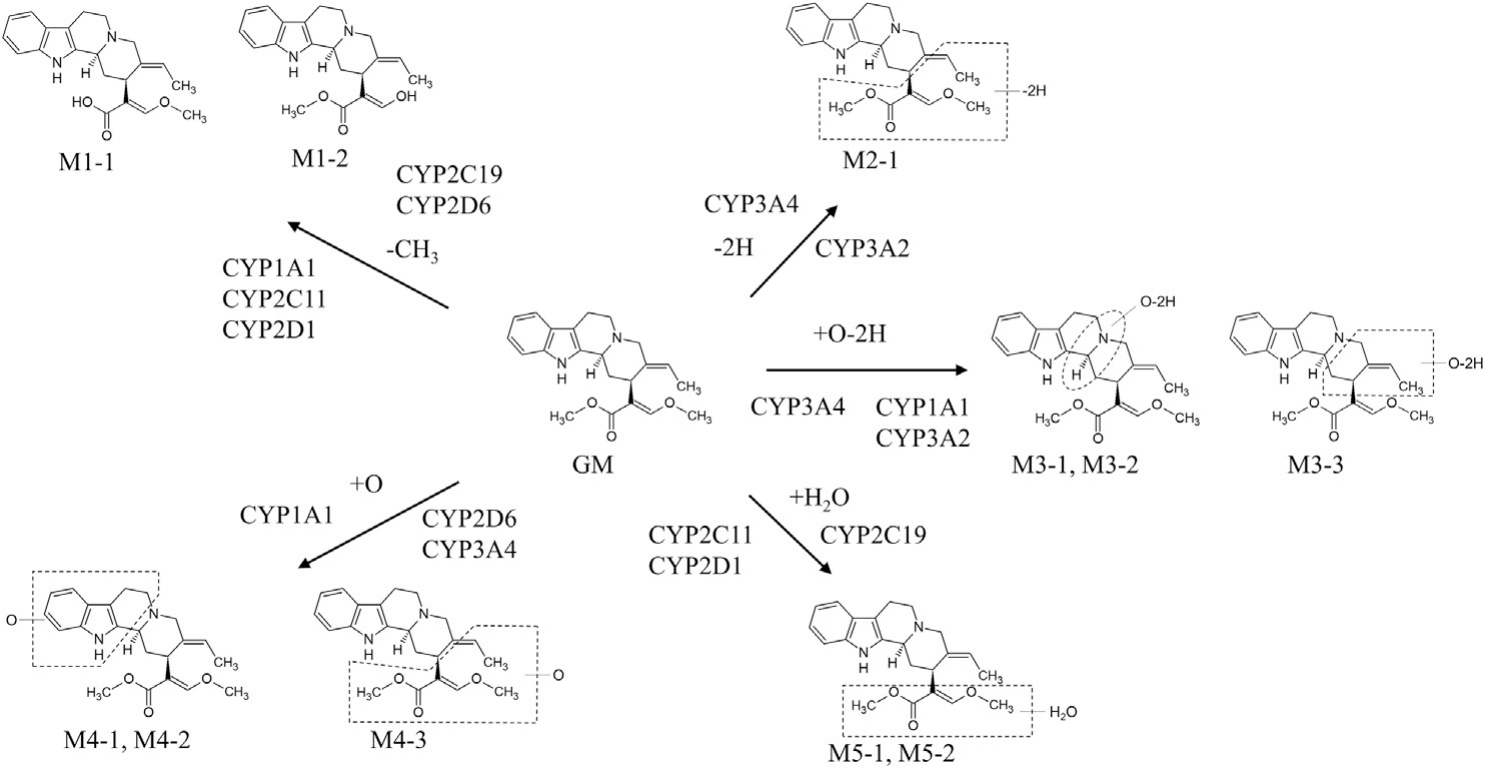
*In vitro* metabolic pathways of GM in rat and human microsomes. The metabolic pathway of GM is the same in humans and rats (Kushida et al., 2015). However, the CYP isoforms involved in each metabolic pathway differ between humans and rats (Matsumoto et al., 2016; Kushida et al., 2021). In humans, GM was dehydrogenated and/or hydroxylated by CYP3A4, demethylated or hydrated by CYP2C19, and demethylated or hydroxylated by CYP2D6. Of these CYP isoforms, CYP3A4-mediated hydroxylation is the major pathway of GM metabolism (Matsumoto et al., 2016). In rats, GM was dehydrogenated and/or hydroxylated by CYP3A2, demethylated and dehydrogenated and/or hydroxylated by CYP1A1, demethylated, hydroxylated, and hydrated by CYP2C11, and demethylated and hydrated by CYP2D1 (Kushida et al., 2021).

More recently, we investigated the CYP isoforms involved in GM liver metabolism in males and females to elucidate the cause of gender differences of rat plasma GM pharmacokinetics (Kushida et al., 2021). When GM was reacted with the rat liver S9 fraction, the reduction of GM was more striking in the male S9 fraction (69.3%) than in the female S9 fraction (10.0%). Screening tests using recombinant rat CYP isoforms illustrated that CYP1A1, CYP2C6, CYP2C11, CYP2D1, and CYP3A2 were involved in GM metabolism. Of these CYP isoforms, male-dependent CYP2C11 and CYP3A2 were found to be predominantly involved in liver microsomal GM metabolism in experiments using anti-rat CYP antibodies. Although the GM metabolic pathway is the same in humans and rats (Kushida et al., 2015), the CYP isoforms involved in their metabolic pathways differ (Figure 2). In humans, GM was dehydrogenated and/or hydroxylated by CYP3A4, demethylated or hydrated by CYP2C19, and demethylated or hydroxylated by CYP2D6. These CYP isoforms reportedly exhibit no gender differences (Martignoni et al., 2016). Of these CYP isoforms, CYP3A4-mediated hydroxylation is the major GM metabolic pathway (Matsumoto et al., 2016). However, in rats, CYP1A1, CYP2C6, CYP2C11, CYP2D1, or CYP3A2 are involved in GM metabolism. Of these CYP isoforms, CYP1A1, CYP2C6, and CYP2D1 are gender-independently expressed in the liver, whereas CYP2C11 and CYP3A2 are male-specific (Martignoni et al., 2016). The demethylation reaction for producing the GM metabolites M1-1/2 was more active in male rats than in females, mainly controlled by CYP2C11 in males and CYP2D1 in females. Similarly, the M2-1–producing dehydrogenation response mainly by CYP3A2 (males) and CYP1A1 (females), the M3-1/3-producing oxidation/dehydrogenation response by CYP3A2 (males) and CYP1A1 (females), and the M5-1/2-producing water addiction reaction by CYP2C11 (males) and CYP2D1 (females) were more dominant in male rats than in female rats. Regarding the M3-2-producing oxidation/dehydrogenation and M4-1/2/3-producing oxidation reactions, CYP1A1 is involved in both sexes, and no gender-related difference could be observed in the case of these reactions. These results suggest that the cause of gender differences in plasma GM pharmacokinetics in rats is most likely because of male-dependent CYP2C11 and CYP3A2 activity. The findings should be useful for interpreting the pharmacological and toxicological effects of GM in the future.

CYP isoforms involved in the metabolism of ICX, RP, IRP, HTE, and HTI have been investigated (Nakazawa et al., 2006; Wang et al., 2010a; Wang et al., 2010b; Zhao et al., 2016b). Zhao et al. (2016b) estimated CYP isoforms involved in the production of the major metabolites of ICX, namely 18,19-dehydrocorynoxinic acid and 5-oxo ICX, using recombinant human CYP isoforms. They found that CYP2C19 and CYP2D6 were involved in the production of 18,19-dehydrocorynoxinic acid, and CYP3A4 was involved in the production of 5-oxo ICX. CYP isoforms involved in the 10- and 11-hydroxylation of RP and IRP have also been inferred from studies using specific inhibitors in rat liver microsomes (Wang et al., 2010a; Wang et al., 2010b). The 10- and 11-hydroxylation of RP and IRP were inhibited by the addition of the specific inhibitors α-naphthoflavone (1A1/2), cimetidine (CYP2C), and quinine (CYP2D), but not by erythromycin (CYP3A). These results suggest that CYP1A1/2, CYP2C, and CYP2D, but not CYP3A, are involved in the 10- and 11-hydroxylation of RP and IRP. Similar inhibition studies using rat liver microsomes also suggested that CYP2C is involved in the 10- and 11-hydroxylation of HTI and HTE (Nakazawa et al., 2006).

### Distribution

#### Exposure to Brain

In addition to plasma, GM has also been detected in the brains of rats orally administered YKS (Imamura et al., 2011; Kushida et al., 2013). Only GM was detected in the brains of male rats orally administered 4 g/kg of YKS. *C*_max_ and *t*_max_ of GM were 1.18 ng/g and 0.5 h, respectively (Kushida et al., 2013). The GM concentrations in the brains of female rats 1 h after the oral administration of 1 and 4 g/kg of YKS were 1.6 and 5.9 ng/g, respectively (Imamura et al., 2011). Concerning other crude drug products, only RP (*C*_max_, 9.6 ng/g; *t*_max_, 2 h) has been detected in the brains of rats orally administered a high dose (25 g/kg) of Gouteng-Baitouweng (Tian et al., 2018).

It has been confirmed that some UH alkaloids can be transferred into the brain when pure drugs are administered (Wang et al., 2010b; Wang et al., 2016b; Wang et al., 2016 W; Zhang C. et al., 2019). CX was detected at a concentration of 11.8 ng/g (3.08 × 10^–11^ mol/g) in the brains of rats after oral administration at a dose of 40 mg/kg (0.105 mol/kg) (Wang W. et al., 2016). RP and IRP have also been detected in the brains of rats with *C*_max_ values of 16.96 and 7.35 ng/g, respectively, after oral administration at a dose of 20 mg/kg (Zhang C. et al., 2019). Wang et al. (2010a) and Wang et al. (2010b) detected RP (0.650 ng/g) in the brains of rats orally administered RP (37.5 mg/kg). HTI was also identified in the rat brain after oral administration at a dose of 20 mg/kg (Wang et al., 2016b).

#### BBB Permeability

Furthermore, an *in vitro* BBB assay using a co-culture of primary rat brain endothelial cells, pericytes, and astrocytes (Nakagawa et al., 2007; Nakagawa et al., 2009) illustrated that the BBB permeability (27.3%) of GM was approximately half that (52.0%) of the antiepileptic drug carbamazepine but higher than those (2.8–16.0%) of six *Uncaria* alkaloids, namely HTE, HTI, RP, IRP, CX, and ICX (Imamura et al., 2011). The BBB permeability of these six alkaloids has also been described in the MDCK-pHaMDR cell monolayer model (Zhang et al., 2017). These six alkaloids also permeabilized the BBB, as reported by Imamura et al. (2011). Furthermore, an examination of BBB permeability using verapamil, a classical P-gp inhibitor, suggested that ICX, IRP, HTE, and HTI could cross the BBB, mainly by passive diffusion, whereas CX and RP cross the BBB *via* facilitated diffusion mediated by the excretion of P-gp.

These *in vivo* and *in vitro* findings suggest that plasma GM, along with other alkaloids, reaches the brain after passing through the BBB. However, the *in vitro* competitive binding assay of seven UH-derived alkaloids to 5-HT_1A_ receptors showed that the IC_50_ value of GM for [^3^H]8-OH-DPAT was 0.904 µM, whereas the other alkaloids either had no competitive binding activity or exhibited IC_50_ values beyond 100 µM (Nishi et al., 2012). Since the *in vitro* GM concentration was far from that in the brain (approximately 100-fold higher), the results of pharmacokinetic studies might not fully explain the pharmacological effects of GM. However, YKS or GM reportedly exhibit the pharmacological effects of improving aggressive behavior and restoring social behavior in mice treated with 1 g/kg of YKS or 150 μg/kg of GM, equivalent to 1 g/kg of YKS extract, respectively (Nishi et al., 2012).

#### Brain Distribution

In the previous sections, we suggested that oral GM definitely enters blood and reaches the brain *via* the BBB. In addition to this evidence, an *in vitro* autoradiography assay using [3H]GM revealed that GM specifically bound to various regions in normal brain slices; i.e., [^3^H]GM bound to dopamine D_2_, adrenergic α_2A_, and μ-opioid receptors; L-type Ca^2+^ channels; and 5-HT_1A_, 5-HT_2A_, 5-HT_2B_, 5-HT_2C_, and 5-HT_7_ receptors (Mizoguchi et al., 2014). Unfortunately, direct *in vivo* evidence of the distribution of GM in the brain remains to be produced. However, we recently demonstrated the brain distributions of GM and its hydroxylated metabolite (HM) in rats intravenously administered GM using DESI–MSI (Matsumoto et al., 2020), a powerful tool for visualizing the distributions of biomolecules in tissue sections (Fernandes et al., 2016). The ion images of GM in the brain sections demonstrated that GM was diffusely distributed throughout the brain parenchyma, including the cerebral cortex, hippocampus, striatum, thalamus, amygdala, and cerebellum. The combined results of DESI–MSI (Matsumoto et al., 2020) and autoradiography (Mizoguchi et al., 2014) suggest that GM is evenly distributed in brain regions and that it exerts pharmacological effects by binding to various channels and receptors. Furthermore, GM signals were detected in the cerebral ventricle even after the parenchymal signals had already disappeared. Contrarily, HM, the main metabolite of GM (Kushida et al., 2015; Matsumoto et al., 2016), was detected only in the ventricle after GM injection and not found in the brain parenchyma, suggesting that plasma HM may have entered cerebrospinal fluid (CSF) directly *via* the blood–CSF barrier (Strazielle and Ghersi-Egea, 2013) and not through the BBB (Laksitorini et al., 2014). CSF is produced in the choroid plexus, and it circulates in the cerebral ventricles. In addition, CSF is absorbed into the venous blood *via* arachnoid granulation, and it protrudes into the dural venous sinuses (Grzybowski et al., 2006). Therefore, it is assumed that GM and HM in CSF are also eventually excreted into venous blood through this system. The *in vivo* evidence of the distribution of GM in the brain (Matsumoto et al., 2020) strongly supports the hypothesis (Ikarashi and Mizoguchi, 2016; Ikarashi et al., 2018) that GM in plasma after the oral administration of YKS enters the brain *via* the BBB and distributes in various brain regions involved in behavioral and psychological symptoms.

As mentioned above, the distribution of GM in the brain, which is the target organ of GM, has been studied, but the transfer and distribution of GM and its metabolites to other organs have not been studied yet. It is necessary to investigate the organ migration and distribution of GM and its metabolites in order to better understand the safety and efficacy of GM. Therefore, it is expected that organ distribution studies of GM will be conducted in the future.

#### Exposure in Other Organs

Exposure to some UH alkaloids, including GM, in tissues other than brain has been investigated (Wang W. et al., 2016; Tian et al., 2018). The pharmacokinetic parameters of CX, ICX, RP, IRP, and GM in liver tissue following oral Gouteng-Baitouweng administration to rats at dose of 25 g/kg were calculated as follows: AUC, 7,149.8, 248.6, 205.8, 4,295.8, and 210.6 ng h/g, respectively, *C*_max_, 1,479.8, 16.1, 52.8, 764.9, and 29.1 ng/g, respectively; and *t*_max_, 1.0, 1.0, 1.0, 1.0, and 1.0 h, respectively (Tian et al., 2018). In another study using pure CX, CX concentrations in the liver, heart, and kidneys 3 h after oral administration (0.105 mol/kg) in rats were 6.38, 8.37, and 12.3 × 10–11 mol/g, respectively (Wang W. et al., 2016). Studying the exposure and time course of UH alkaloids in various organs will provide information on the accumulation of these components and contribute to clarification of the safety of UH alkaloid-containing crude drug products.

### Excretion

#### Urinary Excretion

Table 4 showed the excretion of UH alkaloids in urine and bile. UH alkaloids Two papers on the urinary excretion of GM have been reported. Gai et al. (2014) detected GM and its metabolite 18-hydroxy-GM in 8 h urine samples from mice orally administered YGS (9.1 g/kg). Matsumoto et al. (2018) detected GM in urine samples collected during 24 h from mice orally administered YKS (1 g/kg) in a basic study of drug–drug interactions between memantine and YKS. Furthermore, Gai et al. (2014) confirmed the urinary excretion of CX, ICX, RP, IRP, HTE, 11-hydroxy HTE, and 19-carbonyl HTI in addition to GM in mice orally treated with YGS.

**TABLE 4:** Excretion studies of UH alkaloids.

Study type	Drug/Route/Animal	Detected compound	References
Urinary excretion	YGK (9.1 g/kg), oral, mice	GM and its metabolite 18-hydroxy-GM were detected in 8 h urine samples	Gai et al. (2014)
	YKS (1 g/kg), oral, mice	GM was detected in urine samples collected during 24 h	Matsumoto et al. (2018)
	ICX (40 mg/kg), oral, rats	12 metabolites: 11-hydroxy ICX, 5-oxoisocorynoxeinic acid-22-O-β-D-glucuronide, 10-hydroxy ICX, 17-O-demethyl-16,17-dihydro-5-oxo ICX, 5-oxoisocorynoxeinic acid, 21-hydroxy-5-oxo ICX, oxireno[18, 19]-5-oxo ICX, 18,19-dehydrocorynoxinic acid, 18,19-dehydrocorynoxinic acid B, CX, ICX-N-oxide, and CX-N-oxide	Qi et al. (2015)
	ICX (40 mg/kg), oral, rats	33 metabolites produced by hydrolysis, oxidation, isomerization, demethylation, epoxidation, reduction, glucuronidation, hydroxylation, and N-oxidation	Zhao et al. (2016a)
	IRP (37.5 mg/kg), oral, rats	10- and 11-hydroxylated metabolites of IPR	Wang et al. (2010a)
	RP (37.5 mg/kg), oral, rats	10- and 11-hydroxylated metabolites of RP	Wang et al. (2010b)
	IRP, oral, rats	47 metabolites produced by dehydrogenation, oxidation, hydrolysis, reduction, demethylation, hydroxylation and glucuronide conjugation	Wang et al. (2016a)
	HTE and HTI (50 mg/kg), oal, rats	10- and 11-hydroxylated metabolites and 10- and 11-O-β-D-glucuronide conjugates	Nakazawa et al. (2006)
Biliary excretion	Tianma-Gouteng Yin (2.5 and 5 g/kg), oral, rats	GM, CX, ICX, RP, IRP, HTE, and HTI	Zhan et al. (2017)
	CX (0.105 mM/kg), oral, rats	10-hydroxylated/10-O-β-D-glucuronide conjugates and 11-hydroxylated/11-O-β-D-glucuronide conjugates	Wang et al. (2014a) and Wang et al. (2014b)
	ICX (40 mg/k), oral, rats	10-hydroxylated/10-O-β-D-glucuronide conjugates and 11-hydroxylated/11-O-β-D-glucuronide conjugates	Chen et al. (2014)
	IRP (37.5 mg/kg), oral, rats	10-hydroxylated/10-O-β-D-glucuronide conjugates and 11-hydroxylated/11-O-β-D-glucuronide conjugates	Wang et al. (2010a)
	RP (37.5 mg/kg), oral, rats	10-hydroxylated/10-O-β-D-glucuronide conjugates and 11-hydroxylated/11-O-β-D-glucuronide conjugates	Wang et al. (2010b)
	HTE and HTI (50 mg/kg), oal, rats	10-hydroxylated/10-O-β-D-glucuronide conjugates and 11-hydroxylated/11-O-β-D-glucuronide conjugates	Nakazawa et al. (2006)

As mentioned in the “Metabolism” sections, many metabolites of UH alkaloids are excreted in the urine and bile of rats and mice. Qi et al. (2015) identified 11-hydroxy ICX, 5-oxoisocorynoxeinic acid-22-*O*-*β*-D-glucuronide, 10-hydroxy ICX, 17-*O*-demethyl-16,17-dihydro-5-oxo ICX, 5-oxoisocorynoxeinic acid, 21-hydroxy-5-oxo ICX, oxireno[18, 19]-5-oxo ICX, 18,19-dehydrocorynoxinic acid, 18,19-dehydrocorynoxinic acid B, CX, ICX-N-oxide, and CX-N-oxide in rat urine following oral ICX administration. In another study, 33 metabolites produced by hydrolysis, oxidation, isomerization, demethylation, epoxidation, reduction, glucuronidation, hydroxylation, and N-oxidation were detected in the urine of rats orally treated with ICX (Zhao et al., 2016a). The 10- and 11-hydroxylated metabolites of RP and IPR have been detected in the urine of orally treated rats (Wang et al., 2010a; Wang et al., 2010b), and in other research, 47 metabolites of IRP were identified in the urine of rats (Wang J. et al., 2016). HTI and HTE were also excreted in rat urine as 10- and 11-hydroxylated metabolites and 10- and 11-*O*-*β*-D-glucuronide conjugates (Nakazawa et al., 2006).

#### Biliary Excretion

Zhan et al. (2018) studied UH alkaloids excreted in the bile of rats orally treated with 2.5 and 5 g/kg Tianma-Gouteng Yin granules. The major absorbed components were GM, CX, ICX, RP, IRP, HTE, and HTI in bile.

Similar UH alkaloid metabolites have been identified in urine and bile. Characteristically, the glucuronide conjugates of hydroxylated UH alkaloid metabolites are excreted in bile. For example, CX and ICX are excreted as 10-hydroxylated/10-*O*-*β*-D-glucuronide conjugates and 11-hydroxylated/11-*O*-*β*-D-glucuronide conjugates in the bile of orally treated rats (Wang W. et al., 2014; Chen et al., 2014). Similar glucuronide metabolites have been identified and isolated from rat bile following the oral administration of RP, IRP, HTI, and HTE (Nakazawa et al., 2006; Wang et al., 2010a; Wang et al., 2010b). Although the biliary metabolites of GM have not been investigated, GM is metabolized to 10- and 11-hydroxylated metabolites, similarly as observed for other UH alkaloids (Kushida et al., 2015). Therefore, it is suggested that GM is also converted to 10- and 11-hydroxylated metabolites and then excreted as 10- and 11-*O*-*β*-D-glucuronide conjugates.

## Discussion and Perspectives

The content of UH alkaloids in crude drug is not very high, and is even lower in the extracts of crude drug product (Nishi et al., 2012; Hou et al., 2018; Kushida et al., 2021). In addition, due to the unique properties of crude drug products, such as the pharmacokinetics of multicomponent and the low bioavailability of components, the unchanged components that appear in the circulating blood may be very low. Recent improvements in analytical techniques, especially in the sensitivity of mass spectrometers, have made it possible to quantify extremely low amounts of UH components in blood.

The LC–MS/MS methods for UH alkaloids are more sensitive than HPLC, with a wide linear range for the quantification of UH alkaloids (Table 1). For example, as discussed in the “Exposure to Blood” section, GM plasma concentration levels after oral administration of UH-containing crude drug products have been on the order of ng/ml. As the lower limit of quantification for GM using HPLC is approximately 0.5 μg/ml, this method is not suitable for GM pharmacokinetic studies. The HPLC method seems to be suitable for the analysis of extracts with high content compounds, such as UH-containing herbs or crude drug products. However, the LC–MS/MS lower limit of quantification in the plasma is 0.025–0.2 ng/ml, which is optimal for GM pharmacokinetic studies. However, mass spectrometry sensitivity is known to be affected by the matrix of the sample; in the GM LC–MS/MS analytical method, the rat plasma matrix had little effect on its analytical sensitivity (Kushida et al., 2013; Tian et al., 2018), although the rat brain matrix exhibited a signal suppression of 53.0–55.5% to the GM analysis (Kushida et al., 2013). When analyzing GM in the brain tissue or that of other organs, an appropriate pretreatment method (e.g., solid-phase extraction) should be used to avoid the matrix effect. The MSI method using MALDI and DESI are very useful tools for visualizing the tissue distribution of components in herbs or crude drug products. MALDI–MSI and DESI–MSI ionize target compounds by applying laser and charged solvent spray, respectively. Therefore, the sensitivity of GM detection by MALDI–MSI and DESI–MSI is lower than that of LC–MS/MS, and a large overdose compared to the normal dose is required to detect GM in the tissues. Most recently, an analytical technology has been developed with higher sensitivity than that of the conventional MALDI, which irradiates a second laser (Post-ionization laser). Such further breakthroughs in analytical techniques are essential to study the tissue distribution of GM at healthy doses.

One approach to investigating the absorption of a drug is to calculate its bioavailability by comparing the pharmacokinetic parameters between oral and intravenous administration. Chen et al. (2020) determined the bioavailability of six UH alkaloids, excluding GM, in mice. In another study, the bioavailability of HTI and HTE in rats was obtained (Han et al., 2019). The bioavailabilities of HTI and HTE (4.4 and 8.21%) in mice were extremely different from those (51.0%, and 68.9%) in rats, which displayed relatively low levels. This means that some UH alkaloids undergo different pharmacokinetic changes in different species. As discussed in the “*Metabolism by CYPs in the Liver*” section, male-specific CYP isoforms are involved in the metabolism of UH alkaloids in rats, and the plasma concentration of these agents are lower in males than in females (Kushida et al., 2021). In addition, gender-specific CYP isoforms are also expressed in the intestinal tract of rats, but they have not been identified in mice (Martignoni et al., 2016). Furthermore, the absorption and excretion of drugs may involve transporter proteins that are expressed in various organs such as the intestine, liver, and brain. For example, PEPT1 and OATP2B1 as influx transporter and P-gp and BCRP as efflux transporter were expressed in intestinal epithelial cells, and OATP1B1 and 1B3 as influx transporter and MRP2 as efflux transporter were expressed in hepatocytes, respectively (Nakanishi and Tamai, 2015). It is also possible that the ability of transporters involved in the absorption and excretion of UH alkaloids may differ between rats and mice. These results suggest that the low bioavailability of UH alkaloids in rats are probably attributable to intestinal metabolic and absorption process. GM has not been investigated for bioavailability to date. The identification of metabolic enzymes and drug transporters would be useful for predicting drug–drug interactions. As discussed in the “Metabolism by CYPs in the Liver” section, the metabolic enzymes of UH alkaloids including GM have been identified, whereas their transporters have not been elucidated. It is difficult to predict whether transporters are involved in UH alkaloid uptake and excretion from the currently available data. Future strategies are needed to investigate the absorption of UH alkaloids, including their intestinal membrane permeability and efflux, and the involvement of transporters.

Because crude drug products are complex products consisting of two or more crude drugs, a single crude drug product may contain multiple components with different structures. These structures can affect the properties of the coexisting components through physical and chemical interactions. Hence, the results of pharmacokinetic studies using crude drug products may differ significantly from those using pure drugs. In a pharmacokinetic study of rats administered YGS, MRT, CL/F, and V/F were 7.25 h, 2.72 L h/kg, and 39.79 L/kg, respectively, for RP and 7.82 h, 6.65 L h/kg, and 83.73 L/kg, respectively, for ICX (Gai et al., 2014). Comparing these results with those of a pharmacokinetic study using pure drug-administered mice (Chen et al., 2020), MRTs of RP and ICX in YGS were approximately 5-fold longer than those of the pure drug (1.7 and 1.6 h, respectively), and CL/F and V/F (11.6 and 8.2 L h/kg; 60.1 and 45.7 L/kg) were approximately 3–4-fold smaller. Furthermore, considering the approximately 10-fold greater bioavailability in mice than in rats, CL/F and V/F of YGS were 30–40-fold smaller. These results support the pharmacokinetic differences between crude and pure drug formulations. However, there is a lack of evidence to confirm this phenomenon. It is expected that further research will be conducted to determine the cause of the differences in pharmacokinetics between crude drug products and pure drugs.

Traditional Japanese Kampo medicines are clinically administered between meals. This might explain why research on the impact of food on the efficacy of Kampo medicines, including crude UH-containing drug products such as YKS and YKSCH, and the absorption of their ingredients is scarce. Because GM is an alkaloid with a LogP of 3.445 and pKa of 8.25, it is expectedly absorbed in the weakly alkaline small intestine but not in the strongly acidic stomach. In fact, the GM absorption time was relatively short, displaying a *t*_max_ of 1–2 h. Meals could affect pH changes in the stomach and the subsequent elimination of GM. Therefore, the absorption of GM is potentially influenced by meals. Other UH alkaloids may also be influenced by meals due to their similar physical properties.

Previous metabolic studies of GM have been conducted only in the liver, suggesting that CYP1A, CYP2C, CYP2D, and CYP3A isoforms are involved in the metabolism of GM. CYP3A isoforms are also known to be present in the intestinal tract. As GM is administered orally, the intestinal tract may be the first-pass organ. Therefore, CYP3A expressed in the intestinal tract might be responsible for the observed gender-related GM plasma concentration differences in rats. CYP3A is only involved in dehydrogenation and dehydrogenation/hydroxylation in rats. Assuming that the main metabolic pathway in rats is hydroxylation as in humans, which is probably highly likely, the contribution of CYP3A to the gender-related differences might not be that high, since CYP1A1 is responsible for hydroxylation in rats. However, GM intestinal metabolism has not yet been investigated. Detailed studies on GM metabolism in the intestine, including the identification of CYPs involved in metabolism, contribution ratio to metabolism, and metabolic stability, would probably clarify the gender-related differences and major organs involved in GM metabolism in rats.

As mentioned in the “Metabolism” sections, UH alkaloids are converted into various metabolites such as hydroxylated and demethylated metabolites. Among the metabolic pathways of these metabolites, the main metabolic pathways are probably 10- and 11-hydroxylation followed by 10- and 11-*O*-*β*-D-glucuronide conjugation, as metabolites produced by these reactions were isolated from the urine, feces, and bile of rats administered UH alkaloids. CYP1A1/2, CYP2C, and CYP2D are involved in this hydroxylation in rats, and CYP3A4 and CYP2D6 participate in this process in humans. In addition, CYP2C11 and CYP3A2 have been revealed to participate in demethylation and dehydrogenation based on the results of identification studies of rat CYP isoforms related to GM metabolism. These rat CYP isoforms are expressed in a male-specific manner, which results in gender differences in circulating GM concentrations in rats. Although no gender differences in the rat blood concentrations of UH alkaloids other than GM have been reported till date, gender differences in the blood concentrations of UH alkaloids are expected to occur in rats. Information on the metabolites of UH alkaloids and the enzymes involved in their metabolism will be useful for further pharmacological studies of UH-containing crude drug products and the prediction of drug–drug interactions in clinical use.

When the clinical doses of YKS were orally administered to rats, RP, HTI, HTE, and GM were detected in circulating plasma, and only GM was detected in the brain (Kushida et al., 2013). In *in vivo* studies using pure alkaloids such as CX, RP, IRP and HTI, these were also detected in the brain (Wang et al., 2010b; Wang et al., 2016b; Wang et al., 2016 W; Zhang C. et al., 2019). However, these doses are 20–40 mg/kg, which is 200–1000-fold higher than the content in YKS (Nishi et al., 2012). Thus, UH alkaloids, excluding GM, can be detected in the brain when administered at doses 200–1000-fold higher than the clinical dose, but they are undetectable when the dose of the crude drug products is close to the clinical dose. In addition, YKS or GM reportedly exhibit the pharmacological effects of improving aggressive behavior and restoring social behavior in mice treated with 1 g/kg of YKS or 150 μg/kg of GM, equivalent to 1 g/kg of YKS extract, respectively (Nishi et al., 2012). These results suggest that GM is probably the main pharmacological component of UH-containing crude drug products at clinical doses. However, since the effective concentration of GM in *in vitro* studies was far from GM concentration in the brain (approximately 100-fold higher) (Nakagawa et al., 2012; Nishi et al., 2012; Kanno et al., 2014; Ishida et al., 2016), the results of pharmacokinetic studies might not fully explain the pharmacological effects of GM. GM was shown to be converted to metabolites, such as oxidized and demethylated metabolites. Hydroxylated metabolites have been identified as GM metabolites in the brain (Matsumoto et al., 2020), although other metabolites in the brain have not been investigated yet. Furthermore, whether GM metabolites exhibit a pharmacological activity has not yet been investigated. However, details regarding the brain exposure and distribution of UH alkaloid metabolites and their pharmacological activities have not yet been investigated, and these activities may be responsible for the pharmacological effects. Therefore, relevant studies are needed in the future. Future identification of UH alkaloids metabolites in the brain and their pharmacological studies would help to resolve the discrepancy between pharmacological and brain concentrations.

## Conclusion

In recent years, the development of analytical techniques has permitted pharmacokinetic studies of UH-containing alkaloids. Among the UH-containing alkaloids, GM is an alkaloid with strong pharmacological activity. GM appears rapidly in the blood after oral administration and passes through the BBB to the target organ, the brain. GM is metabolized by hepatic CYP isoforms to metabolites such as demethylated, dehydrogenated, and hydroxylated forms. Other UH alkaloids follow the same *in vivo* fate as GM. Observation of the pharmacokinetics of UH alkaloids is extremely important for understanding the pharmacological activity, efficacy, and safety of UH-containing drug treatments. However, although pharmacokinetic studies of UH alkaloids have progressed, they are inadequate, and further studies are needed to clarify certain issues, such as the effects of food on UH alkaloid absorption, the excretion profile of the metabolites of the alkaloids, their metabolism in intestinal tissues, and differences in pharmacokinetics between crude drug products and pure drugs. These aspects would potentially provide a better understanding of UH-containing crude drug product efficacy and safety.

## References

[B1] Chen L.-L. Song J.-X. Lu J.-H. Yuan Z.-W. Liu L.-F. Durairajan S. S. K. (2014). Corynoxine, a Natural Autophagy Enhancer, Promotes the Clearance of Alpha-Synuclein via Akt/mTOR Pathway. J. Neuroimmune Pharmacol. 9, 380–387. 10.1007/s11481-014-9528-2 24522518

[B2] Chen L. Ma J. Wang X. Zhang M. (2020). Simultaneous Determination of Six Uncaria Alkaloids in Mouse Blood by UPLC-MS/MS and its Application in Pharmacokinetics and Bioavailability. Biomed. Res. Int. 2020, 1–11. 10.1155/2020/1030269 PMC7448256 32879877

[B3] Chua K. K. Chau C. C. Li M. (2012). Experimental and Clinical Research Literature Review of Tianma Gouteng Yin on the Treatment of Parkinson’s Disease. Hong Kong J. Tradi. Chin. Med. 7, 66–70.

[B4] Egashira N. Mishima K. Kurauchi K. Iwasaki K. Fujiwara M. (2001). Cholinergic Involvement in the Improving Effects of Yoku-Kan-San-Ka-Chimpi-Hange (Yi-Gan-San- Jia-Chen-Pi-Ban-Xia) on the Disruption of Spatial Cognition and the Electroconvulsive Shock-Induced Immobilization. J. Trad. Med. 18, 71–80.

[B5] Fernandes A. M. A. P. Vendramini P. H. Galaverna R. Schwab N. V. Alberici L. C. Augusti R. (2016). Direct Visualization of Neurotransmitters in Rat Brain Slices by Desorption Electrospray Ionization Mass Spectrometry Imaging (DESI - MS). J. Am. Soc. Mass. Spectrom. 27, 1944–1951. 10.1007/s13361-016-1475-0 27704473

[B6] Fu A. K. Y. Hung K.-W. Huang H. Gu S. Shen Y. Cheng E. Y. L. (2014). Blockade of EphA4 Signaling Ameliorates Hippocampal Synaptic Dysfunctions in Mouse Models of Alzheimer's Disease. Proc. Natl. Acad. Sci. USA. 111, 9959–9964. 10.1073/pnas.1405803111 24958880 PMC4103318

[B7] Gai Y. Chen H. Liu W. Feng F. Xie N. (2014). The Metabolism of YiGan San and Subsequent Pharmacokinetic Evaluation of Four Metabolites in Rat Based on Liquid Chromatography with Tandem Mass Spectrometry. J. Chromatogr. B 972, 22–28. 10.1016/j.jchromb.2014.09.033 25306115

[B8] Giordano S. Zucchetti M. Decio A. Cesca M. Fuso Nerini I. Maiezza M. (2016). Heterogeneity of Paclitaxel Distribution in Different Tumor Models Assessed by MALDI Mass Spectrometry Imaging. Sci. Rep. 6, 39284. 10.1038/srep39284 28000726 PMC5175283

[B9] Grzybowski D. M. Holman D. W. Katz S. E. Lubow M. (2006). *In Vitro* model of Cerebrospinal Fluid Outflow through Human Arachnoid Granulations. Invest. Ophthalmol. Vis. Sci. 47, 3664–3672. 10.1167/iovs.05-0929 16877441

[B10] Han A. Lin G. Cai J. Wu Q. Geng P. Ma J. (2019). Pharmacokinetic Study on Hirsutine and Hirsuteine in Rats Using UPLC-MS/MS. Acta Chromatographica 31, 99–104. 10.1556/1326.2017.00365

[B11] Hou J. Feng R. Zhang Y. Pan H. Yao S. Han S. (2018). Characteristic Chromatogram: a Method of Discriminate and Quantitative Analysis for Quality Evaluation of Uncaria Stem with hooks. Planta Med. 84, 449–456. 10.1055/s-0043-123827 29216668

[B12] Ikarashi Y. Mizoguchi K. (2016). Neuropharmacological Efficacy of the Traditional Japanese Kampo Medicine Yokukansan and its Active Ingredients. Pharmacol. Ther. 166, 84–95. 10.1016/j.pharmthera.2016.06.018 27373856

[B13] Ikarashi Y. Sekiguchi K. Mizoguchi K. (2018). Serotonin Receptor Binding Characteristics of Geissoschizine Methyl Ether, an Indole Alkaloid in Uncaria Hook. Cmc 25, 1036–1045. 10.2174/0929867324666170320114713 PMC5898036 28322152

[B14] Imamura S. Tabuchi M. Kushida H. Nishi A. Kanno H. Yamaguchi T. (2011). The Blood-Brain Barrier Permeability of Geissoschizine Methyl Ether in Uncaria Hook, a Galenical Constituent of the Traditional Japanese Medicine Yokukansan. Cell Mol. Neurobiol. 31, 787–793. 10.1007/s10571-011-9676-3 21442303 PMC11498390

[B15] Ishida Y. Ebihara K. Tabuchi M. Imamura S. Sekiguchi K. Mizoguchi K. (2016). Yokukansan, a Traditional Japanese Medicine, Enhances the L-DOPA-Induced Rotational Response in 6-Hydroxydopamine-Lesioned Rats: Possible Inhibition of COMT. Biol. Pharm. Bull. 39, 104–113. 10.1248/bpb.b15-00691 26725433

[B16] Ishikawa A. Makino K. Idezuka J. Kuwabara T. (2006). Improvement of Symptoms Following Epileptic Convulsion in a Patient with Parkinson's Disease. Mov. Disord. 21, 1055. 10.1002/mds.20885 16622852

[B17] Ito A. Shin N. Tsuchida T. Okubo T. Norimoto H. (2013). Antianxiety-like Effects of Chimpi (Dried Citrus Peels) in the Elevated Open-Platform Test. Molecules 18, 10014–10023. 10.3390/molecules180810014 23966085 PMC6270198

[B18] Jiang P. Chen L. Sun J. Li J. Xu J. Liu W. (2019). Chotosan Ameliorates Cognitive Impairment and hippocampus Neuronal Loss in Experimental Vascular Dementia via Activating the Nrf2-Mediated Antioxidant Pathway. J. Pharmacol. Sci. 139, 105–111. 10.1016/j.jphs.2018.12.003 30642751

[B19] Kanno H. Kawakami Z. Mizoguchi K. Ikarashi Y. Kase Y. (2014). Yokukansan, a Kampo Medicine, Protects PC12 Cells from Glutamate-Induced Death by Augmenting Gene Expression of Cystine/Glutamate Antiporter System Xc−. PLoS One 9, e116275. 10.1371/journal.pone.0116275 25551766 PMC4281137

[B20] Kawakami Z. Ikarashi Y. Kase Y. (2011). Isoliquiritigenin Is a Novel NMDA Receptor Antagonist in Kampo Medicine Yokukansan. Cel Mol. Neurobiol. 31, 1203–1212. 10.1007/s10571-011-9722-1 PMC11498536 21691759

[B21] Kim T.-J. Lee J.-H. Lee J.-J. Yu J.-Y. Hwang B.-Y. Ye S.-K. (2008). Corynoxeine Isolated from the Hook of Uncaria Rhynchophylla Inhibits Rat Aortic Vascular Smooth Muscle Cell Proliferation through the Blocking of Extracellular Signal Regulated Kinase 1/2 Phosphorylation. Biol. Pharm. Bull. 31, 2073–2078. 10.1248/bpb.31.2073 18981576

[B22] Kitagawa H. Munekage M. Ichikawa K. Fukudome I. Munekage E. Takezaki Y. (2015). Pharmacokinetics of Active Components of Yokukansan, a Traditional Japanese Herbal Medicine after a Single Oral Administration to Healthy Japanese Volunteers: a Cross-Over, Randomized Study. PLoS One 10, e0131165. 10.1371/journal.pone.0131165 26151135 PMC4495062

[B23] Kushida H. Fukutake M. Tabuchi M. Katsuhara T. Nishimura H. Ikarashi Y. (2013). Simultaneous Quantitative Analyses of Indole and Oxindole Alkaloids of Uncaria Hook in Rat Plasma and Brain after Oral Administration of the Traditional Japanese Medicine Yokukansan Using High-Performance Liquid Chromatography with Tandem Mass Spectrometr. Biomed. Chromatogr. 27, 1647–1656. 10.1002/bmc.2974 23813572

[B24] Kushida H. Matsumoto T. Igarashi Y. Nishimura H. Watanabe J. Maemura K. (2015). Metabolic Profiling of the Uncaria Hook Alkaloid Geissoschizine Methyl Ether in Rat and Human Liver Microsomes Using High-Performance Liquid Chromatography with Tandem Mass Spectrometry. Molecules 20, 2100–2114. 10.3390/molecules20022100 25633336 PMC6272236

[B25] Kushida H. Matsumoto T. Ikarashi Y. Nishimura H. Yamamoto M. (2021). Gender Differences in Plasma Pharmacokinetics and Hepatic Metabolism of Geissoschizine Methyl Ether from Uncaria Hook in Rats. J. Ethnopharmacology 264, 113354. 10.1016/j.jep.2020.113354 32898626

[B26] Laksitorini M. Prasasty V. D. Kiptoo P. K. Siahaan T. J. (2014). Pathways and Progress in Improving Drug Delivery through the Intestinal Mucosa and Blood-Brain Barriers. Ther. Deliv. 5, 1143–1163. 10.4155/tde.14.67 25418271 PMC4445828

[B27] Li X.-M. Zhang X.-J. Dong M.-X. (2017). Isorhynchophylline Attenuates MPP+-Induced Apoptosis through Endoplasmic Reticulum Stress- and Mitochondria-dependent Pathways in PC12 Cells: Involvement of Antioxidant Activity. Neuromol Med. 19, 480–492. 10.1007/s12017-017-8462-x 28822073

[B28] Liang J.-H. Wang C. Huo X.-K. Tian X.-G. Zhao W.-Y. Wang X. (2020). The Genus Uncaria: A Review on Phytochemical Metabolites and Biological Aspects. Fitoterapia 147, 104772. 10.1016/j.fitote.2020.104772 33152463

[B29] Liu L.-F. Song J.-X. Lu J.-H. Huang Y.-Y. Zeng Y. Chen L.-L. (2015). Tianma Gouteng Yin, a Traditional Chinese Medicine Decoction, Exerts Neuroprotective Effects in Animal and Cellular Models of Parkinson's Disease. Sci. Rep. 5, 16862. 10.1038/srep16862 26578166 PMC4649620

[B30] Lu J.-H. Tan J.-Q. Durairajan S. S. K. Liu L.-F. Zhang Z.-H. Ma L. (2012). Isorhynchophylline, a Natural Alkaloid, Promotes the Degradation of Alpha-Synuclein in Neuronal Cells via Inducing Autophagy. Autophagy 8, 98–108. 10.4161/auto.8.1.18313 22113202

[B31] Ma B. Wang J. Sun J. Li M. Xu H. Sun G. (2014). Permeability of Rhynchophylline across Human Intestinal Cell *In Vitro* . Int. J. Clin. Exp. Pathol. 7, 957–966. PMC4069913 24966905

[B32] Martignoni M. Groothuis G. M. M. de Kanter R. (2006). Species Differences between Mouse, Rat, Dog, Monkey and Human CYP-Mediated Drug Metabolism, Inhibition and Induction. Expert Opin. Drug Metab. Toxicol. 2, 875–894. 10.1517/17425255.2.6.875 17125407

[B33] Matsumoto K. Zhao Q. Niu Y. Fujiwara H. Tanaka K. Sasaki-Hamada S. (2013). Kampo Formulations, Chotosan, and Yokukansan, for Dementia Therapy: Existing Clinical and Preclinical Evidence. J. Pharmacol. Sci. 122, 257–269. 10.1254/jphs.13R03CR 23883485

[B34] Matsumoto T. Ikarashi Y. Takiyama M. Watanabe J. Setou M. (2020). Brain Distribution of Geissoschizine Methyl Ether in Rats Using Mass Spectrometry Imaging Analysis. Sci. Rep. 10, 7293. 10.1038/s41598-020-63474-x 32350314 PMC7190722

[B35] Matsumoto T. Kushida H. Maruyama T. Nishimura H. Watanabe J. Maemura K. (2016). In Vitroidentification of Human Cytochrome P450 Isoforms Involved in the Metabolism of Geissoschizine Methyl Ether, an Active Component of the Traditional Japanese Medicine Yokukansan. Xenobiotica 46, 325–334. 10.3109/00498254.2015.1076585 26337900

[B36] Matsumoto T. Kushida H. Matsushita S. Oyama Y. Suda T. Watanabe J. (2017). Distribution Analysis via Mass Spectrometry Imaging of Ephedrine in the Lungs of Rats Orally Administered the Japanese Kampo Medicine Maoto. Sci. Rep. 7, 44098. 10.1038/srep44098 28272490 PMC5341069

[B37] Matsumoto T. Sekiguchi K. Kawakami Z. Watanabe J. Mizoguchi K. Ikarashi Y. (2018). Basic Study of Drug-Drug Interaction between Memantine and the Traditional Japanese Kampo Medicine Yokukansan. Molecules 24, 115. 10.3390/molecules24010115 PMC6337661 30597998

[B38] Ministry of Health, Labour, and Welfare MHLW (2016). Japanese Pharmacopoeia 17th Edition: the MHLW Ministerial Notification No. 64. Availableat: https://www.mhlw.go.jp/file/06-Seisakujouhou-11120000-Iyakushokuhinkyoku/JP17_REV.pdf.

[B39] Mizoguchi K. Ikarashi Y. (2017). Cellular Pharmacological Effects of the Traditional Japanese Kampo Medicine Yokukansan on Brain Cells. Front. Pharmacol. 8, 655. 10.3389/fphar.2017.00655 28979206 PMC5611794

[B40] Mizoguchi K. Kushida H. Kanno H. Igarashi Y. Nishimura H. Ikarashi Y. (2014). Specific Binding and Characteristics of Geissoschizine Methyl Ether, an Indole Alkaloid of Uncaria Hook, in the Rat Brain. J. Ethnopharmacology 158, 264–270. 10.1016/j.jep.2014.10.015 25456433

[B41] Murakami Y. Zhao Q. Harada K. Tohda M. Watanabe H. Matsumoto K. (2005). Choto-san, a Kampo Formula, Improves Chronic Cerebral Hypoperfusion-Induced Spatial Learning Deficit via Stimulation of Muscarinic M Receptor. Pharmacol. Biochem. Behav. 81, 616–625. 10.1016/j.pbb.2005.05.004 15936808

[B42] Nakagawa S. Deli M. A. Kawaguchi H. Shimizudani T. Shimono T. Kittel Á. (2009). A New Blood-Brain Barrier Model Using Primary Rat Brain Endothelial Cells, Pericytes and Astrocytes. Neurochem. Int. 54, 253–263. 10.1016/j.neuint.2008.12.002 19111869

[B43] Nakagawa S. Deli M. A. Nakao S. Honda M. Hayashi K. Nakaoke R. (2007). Pericytes from Brain Microvessels Strengthen the Barrier Integrity in Primary Cultures of Rat Brain Endothelial Cells. Cell. Mol. Neurobiol. 27, 687–694. 10.1007/s10571-007-9195-4 17823866 PMC11517186

[B44] Nakagawa T. Nagayasu K. Nishitani N. Shirakawa H. Sekiguchi K. Ikarashi Y. (2012). Yokukansan Inhibits Morphine Tolerance and Physical Dependence in Mice: The Role of α2A-adrenoceptor. Neuroscience 227, 336–349. 10.1016/j.neuroscience.2012.09.079 23069764

[B45] Nakanishi T. Tamai I. (2015). Interaction of Drug or Food with Drug Transporters in Intestine and Liver. Cdm 16, 753–764. 10.2174/138920021609151201113537 26630906

[B46] Nakatani Y. Amano T. Yamamoto H. Sakai N. Tsuji M. Takeda H. (2017). Yokukansan Enhances the Proliferation of B65 Neuroblastoma. J. Traditional Complement. Med. 7, 34–44. 10.1016/j.jtcme.2016.01.006 PMC5198832 28053886

[B47] Nakazawa T. Banba K.-i. Hata K. Nihei Y. Hoshikawa A. Ohsawa K. (2006). Metabolites of Hirsuteine and Hirsutine, the Major Indole Alkaloids of Uncaria Rhynchophylla, in Rats. Biol. Pharm. Bull. 29, 1671–1677. 10.1248/bpb.29.1671 16880624

[B48] Ndagijimana A. Wang X. Pan G. Zhang F. Feng H. Olaleye O. (2013). A Review on Indole Alkaloids Isolated from Uncaria Rhynchophylla and Their Pharmacological Studies. Fitoterapia 86, 35–47. 10.1016/j.fitote.2013.01.018 23376412

[B49] Nishi A. Yamaguchi T. Sekiguchi K. Imamura S. Tabuchi M. Kanno H. (2012). Geissoschizine Methyl Ether, an Alkaloid in Uncaria Hook, Is a Potent serotonin1A Receptor Agonist and Candidate for Amelioration of Aggressiveness and Sociality by Yokukansan. Neuroscience 207, 124–136. 10.1016/j.neuroscience.2012.01.037 22314317

[B50] Pengsuparp T. Indra B. Nakagawasai O. Tadano T. Mimaki Y. Sashida Y. (2001). Pharmacological Studies of Geissoschizine Methyl Ether, Isolated from Uncaria Sinensis Oliv., in the central Nervous System. Eur. J. Pharmacol. 425, 211–218. 10.1016/S0014-2999(01)01195-5 11513840

[B51] Qi W. Chen F. Sun J. Simpkins J. Yuan D. (2015). Isolation and Identification of Twelve Metabolites of Isocorynoxeine in Rat Urine and Their Neuroprotective Activities in HT22 Cell Assay. Planta Med. 81, 46–55. 10.1055/s-0034-1383357 25519834 PMC4461055

[B52] Qi W. Yue S.-J. Sun J.-H. Simpkins J. W. Zhang L. Yuan D. (2014). Alkaloids from the Hook-Bearing branch ofUncariarhynchophyllaand Their Neuroprotective Effects against Glutamate-Induced HT22 Cell Death. J. Asian Nat. Prod. Res. 16, 876–883. 10.1080/10286020.2014.918109 24899363 PMC4446702

[B53] Sakakibara I. Terabayashi S. Kubo M. Higuchi M. Komatsu Y. Okada M. (1999). Effect on Locomotion of Indole Alkaloids from the hooks of Uncaria Plants. Phytomedicine 6, 163–168. 10.1016/S0944-7113(99)80004-X 10439480

[B54] Strazielle N. Ghersi-Egea J. F. (2013). Physiology of Blood-Brain Interfaces in Relation to Brain Disposition of Small Compounds and Macromolecules. Mol. Pharmaceutics 10, 1473–1491. 10.1021/mp300518e 23298398

[B55] Sun J. Ren X. Qi W. Yuan D. Simpkins J. W. (2016). Geissoschizine Methyl Ether Protects Oxidative Stress-Mediated Cytotoxicity in Neurons through the 'Neuronal Warburg Effect'. J. Ethnopharmacology 187, 249–258. 10.1016/j.jep.2016.04.034 PMC4887292 27114061

[B56] Tabuchi M. Mizuno K. Mizoguchi K. Hattori T. Kase Y. (2017). Yokukansan and Yokukansankachimpihange Ameliorate Aggressive Behaviors in Rats with Cholinergic Degeneration in the Nucleus Basalis of Meynert. Front. Pharmacol. 8, 235. 10.3389/fphar.2017.00235 28491038 PMC5405124

[B57] Takiyama M. Matsumoto T. Watanabe J. (2019). LC-MS/MS Detection of Citrus Unshiu Peel-Derived Flavonoids in the Plasma and Brain after Oral Administration of Yokukansankachimpihange in Rats. Xenobiotica 49, 1494–1503. 10.1080/00498254.2019.1581300 30741064

[B58] Tian X. Xu Z. Chen M. Hu P. Liu F. Sun Z. (2018). Simultaneous Determination of Eight Bioactive Compounds by LC-MS/MS and its Application to the Pharmacokinetics, Liver First-Pass Effect, Liver and Brain Distribution of Orally Administrated Gouteng-Baitouweng (GB) in Rats. J. Chromatogr. B 1084, 122–131. 10.1016/j.jchromb.2018.03.013 29597038

[B59] Tsuji M. Takeuchi T. Miyagawa K. Ishii D. Imai T. Takeda K. (2014). Yokukansan, a Traditional Japanese Herbal Medicine, Alleviates the Emotional Abnormality Induced by Maladaptation to Stress in Mice. Phytomedicine 21, 363–371. 10.1016/j.phymed.2013.08.025 24129119

[B60] Ueda T. Ugawa S. Ishida Y. Shimada S. (2011). Geissoschizine Methyl Ether Has Third-Generation Antipsychotic-like Actions at the Dopamine and Serotonin Receptors. Eur. J. Pharmacol. 671, 79–86. 10.1016/j.ejphar.2011.09.007 21951966

[B61] Ueki T. Nishi A. Imamura S. Kanno H. Mizoguchi K. Sekiguchi K. (2013). Effects of Geissoschizine Methyl Ether, an Indole Alkaloid in Uncaria Hook, a Constituent of Yokukansan, on Human Recombinant Serotonin7 Receptor. Cell Mol Neurobiol 33, 129–135. 10.1007/s10571-012-9878-3 22968712 PMC11497864

[B62] Wang H.-B. Qi W. Zhang L. Yuan D. (2014a). Qualitative and Quantitative Analyses of Alkaloids in Uncaria Species by UPLC-ESI-Q-TOF/MS. Chem. Pharm. Bull. 62, 1100–1109. 10.1248/cpb.c14-00481 25366313

[B63] Wang J. Qi P. Hou J. Shen Y. Yan B. Bi Q. (2016a). Profiling and Identification of Metabolites of Isorhynchophylline in Rats by Ultra High Performance Liquid Chromatography and Linear Ion Trap Orbitrap Mass Spectrometry. J. Chromatogr. B 1033-1034, 147–156. 10.1016/j.jchromb.2016.08.013 27561181

[B64] Wang J. Qi P. Hou J. Shen Y. Yang M. Bi Q. (2017b). The Profiling of the Metabolites of Hirsutine in Rat by Ultra-high Performance Liquid Chromatography Coupled with Linear Ion Trap Orbitrap Mass Spectrometry: An Improved Strategy for the Systematic Screening and Identification of Metabolites in Multi-Samples *In Vivo* . J. Pharm. Biomed. Anal. 134, 149–157. 10.1016/j.jpba.2016.11.034 27915192

[B65] Wang W. Li X. Chen Y. Hattori M. (2014b). Structural Elucidation of Rat Biliary Metabolites of Corynoxeine and Their Quantification Using LC-MSn. Biomed. Chromatogr. 28, 1219–1228. 10.1002/bmc.3149 24523045

[B66] Wang W. Luo S. Chen Y. Li B. Hattori M. (2016c). Effective Separation and Simultaneous Determination of Corynoxeine and its Metabolites in Rats by High-Performance Liquid Chromatography with Tandem Mass Spectrometry and Application to Pharmacokinetics and *In Vivo* Distribution in Main Organs. Anal. Sci. 32, 705–707. 10.2116/analsci.32.705 27302594

[B67] Wang W. Ma C.-M. Hattori M. (2010b). Metabolism and Pharmacokinetics of Rhynchophylline in Rats. Biol. Pharm. Bull. 33, 669–676. 10.1248/bpb.33.669 20410604

[B68] Wang W. Ma C.-M. Hattori M. (2010a). Metabolism of Isorhynchophylline in Rats Detected by LC-MS. J. Pharm. Pharm. Sci. 13, 27–37. 10.18433/j33g60 20456828

[B69] Wang X. Zheng M. Liu J. Huang Z. Bai Y. Ren Z. (2017). Differences of First-Pass Effect in the Liver and Intestine Contribute to the Stereoselective Pharmacokinetics of Rhynchophylline and Isorhynchophylline Epimers in Rats. J. Ethnopharmacology 209, 175–183. 10.1016/j.jep.2017.07.039 28755970

[B70] Watanabe H. Zhao Q. Matsumoto K. Tohda M. Murakami Y. Zhang S.-H. (2003). Pharmacological Evidence for Antidementia Effect of Choto-San (Gouteng-San), a Traditional Kampo Medicine. Pharmacol. Biochem. Behav. 75, 635–643. 10.1016/s0091-3057(03)00109-6 12895681

[B71] Xian Y.-F. Fan D. Ip S.-P. Mao Q.-Q. Lin Z.-X. (2017). Antidepressant-like Effect of Isorhynchophylline in Mice. Neurochem. Res. 42, 678–685. 10.1007/s11064-016-2124-5 27900600

[B72] Xian Y.-F. Lin Z.-X. Mao Q.-Q. Hu Z. Zhao M. Che C.-T. (2012). Bioassay-Guided Isolation of Neuroprotective Compounds fromUncaria Rhynchophyllaagainst Beta-Amyloid-Induced Neurotoxicity. Evidence-Based Complement. Altern. Med. 2012, 1–8. 10.1155/2012/802625 PMC3388340 22778778

[B73] Yang W. Ip S.-P. Liu L. Xian Y.-F. Lin Z.-X. (2020). *Uncaria Rhynchophylla* and its Major Constituents on central Nervous System: a Review on Their Pharmacological Actions. Cvp 18, 346–357. 10.2174/1570161117666190704092841 31272356

[B74] Yuzurihara M. Ikarashi Y. Goto K. Sakakibara I. Hayakawa T. Sasaki H. (2002). Geissoschizine methyl ether, an indole alkaloid extracted from Uncariae Ramulus et Uncus, is a potent vasorelaxant of isolated rat aorta. Eur. J. Pharmacol. 444, 183–189. 10.1016/s0014-2999(02)01623-0 12063078

[B75] Zhang C. Wu X. Xian Y. Zhu L. Lin G. Lin Z.-X. (2019a). Evidence on Integrating Pharmacokinetics to Find Truly Therapeutic Agent for Alzheimer's Disease: Comparative Pharmacokinetics and Disposition Kinetics Profiles of Stereoisomers Isorhynchophylline and Rhynchophylline in Rats. Evidence-Based Complement. Altern. Med. 2019, 1–9. 10.1155/2019/4016323 PMC6377964 30854007

[B76] Zhang H. Duan S. Wang L. Liu J. Qi W. Yuan D. (2019b). Identification of the Absorbed Components and Their Metabolites of Tianma-Gouteng Granule in Rat Plasma and Bile Using Ultra-high-performance Liquid Chromatography Combined with Quadrupole Time-Of-Flight Mass Spectrometry. Biomed. Chromatogr. 33, e4480. 10.1002/bmc.4480 30597588

[B77] Zhang Q. Zhao J. J. Xu J. Feng F. Qu W. (2015). Medicinal Uses, Phytochemistry and Pharmacology of the Genus Uncaria. J. Ethnopharmacology 173, 48–80. 10.1016/j.jep.2015.06.011 26091967

[B78] Zhang Y.-N. Yang Y.-F. Xu W. Yang X.-W. (2017). The Blood-Brain Barrier Permeability of Six Indole Alkaloids from Uncariae Ramulus Cum Uncis in the MDCK-pHaMDR Cell Monolayer Model. Molecules 22, 1944. 10.3390/molecules22111944 PMC6150385 29125571

[B79] Zhao L. Qi W. Chen F. Sun J. Yuan D. (2016a). Metabolic Profile of Isocorynoxeine in Rats Obtained by Ultra-high Performance Liquid Chromatography/quadrupole Time-Of-Flight Mass Spectrometry. Eur. J. Drug Metab. Pharmacokinet. 41, 615–626. 10.1007/s13318-015-0287-0 26077124

[B80] Zhao L. Zang B. Qi W. Chen F. Wang H. Kano Y. (2016b). Pharmacokinetic Study of Isocorynoxeine Metabolites Mediated by Cytochrome P450 Enzymes in Rat and Human Liver Microsomes. Fitoterapia 111, 49–57. 10.1016/j.fitote.2016.04.008 27094112

[B81] Zhao Q. Murakami Y. Tohda M. Watanabe H. Matsumoto K. (2005). Preventive Effect of Chotosan, a Kampo Medicine, on Transient Ischemia-Induced Learning Deficit Is Mediated by Stimulation of Muscarinic M1 but Not Nicotinic Receptor. Biol. Pharm. Bull. 28, 1873–1878. 10.1248/bpb.28.1873 16204938

